# Top-down modulation impairs priming susceptibility in complex decision-making with social implications

**DOI:** 10.1038/s41598-022-22707-x

**Published:** 2022-10-25

**Authors:** Franco Agustín Bernal, Tomás Alves Salgueiro, Axel Brzostowski, Emilio Recart Zapata, Ayelén Carames, Juan Manuel Pérez, Damián Furman, Martín Graziano, Pablo Nicolás Fernández Larrosa

**Affiliations:** 1 Instituto de Fisiología, Biología Molecular y Neurociencias (IFIByNE-UBA-CONICET), Buenos Aires, Argentina; 2 Departamento de Computación, Facultad de Ciencias Exactas y Naturales, Universidad de Buenos Aires, Buenos Aires, Argentina; 3 Instituto de Ciencias de la Computación (ICC), Universidad de Buenos Aires, Buenos Aires, Argentina; 4 Instituto de Ecología, Genética y Evolución, Facultad de Ciencias Exactas y Naturales, Universidad de Buenos Aires, Buenos Aires, Argentina

**Keywords:** Molecular neuroscience, Social neuroscience

## Abstract

Could social context variables prime complex decisions? Could top-down processes impair this priming susceptibility? Complex decisions have been mainly studied from economic and moral perspectives, and Dual Process Theories provide evidence of how these processes could be affected. To address these issues from a political perspective, online experiments were conducted. Participants (n = 252) were asked to choose a face from 4 options, each associated with different frequencies (repetition priming) or with phrases with different emotional valence (emotional priming), for an unspecified task (UST group) or an important task (IMT group). The most repeated face was chosen most in the UST group, and was associated with lower response times. Positive faces were equally chosen by both groups. To compare results in a more ecological situation, a social study was conducted during the 2019 Argentine Presidential Election, including online surveys (n = 3673) and analysis of news media mentioning candidates. The familiarity and trust to each candidate explained the voting-probability for most of them, as well as correlated with their frequency of mentions in the news, their positive associations, and election results. Our results suggest complex decision-making is susceptible to priming, depending on top-down modulation.

## Introduction

Decision-making (DM) is a constitutive cognitive process in our daily activities. Most simple decisions are made rapidly and at a low cognitive level, like perceptual or visual search processes^1,2^. Most of the experimental paradigms focusing on this type of DM involve two-choice tasks^1–4^ (for multiple-choice decisions, see^5–7^). Nevertheless, other decisions, such as which candidate to vote for in an election, could require a higher cognitive level and longer reflection^1^. While most decisions are processes of selecting an option from a set of alternatives on the basis of their likelihood of leading to the best possible outcome, some DM would require that they were not made in haste and that decision-makers were aware of the potential outcomes. We conceptualize them as complex decision-making processes. The relevance of this type of DM has caused it to be studied from the perspectives of cognitive psychology to neuroeconomics, providing an amalgam of very heterogeneous studies with diverse approaches including canonical Theory of Games^8,9^, Kahneman’s framework^10,11^, monetary gambles (i.e. Iowa Gambling Task)^12^, and moral dilemmas^13,14^, among others. Most of these frameworks assume some rationality criterion beyond the decision, though Kahneman’s perspective incorporates the idea of relative preference that could be affected by social norms, expectations, or levels of aspiration^10,11^. Dual Process theories support the thesis that DM could be the result of two qualitatively different types of processes^11,15–17^, which differ in the degree to which they are characterised as fast and automatic (Type-1) or slow and deliberate (Type-2). However, recent theories suggest a 'two-stage hybrid model' in which Type-1 and Type-2 processes are defined along a continuum rather than a dichotomy^16,18,19^.

Other frameworks analyse the DM process from a political or social perspective, including studies in the areas of dynamics of opinion change^20–23^, confirmation and social biases^24–27^, confirmation bias^28,29^, self-deception^30–32^, political polarization^23,32–34^, and fake news influence^35–38^, among others. All these studies support thinking of DM as processes that can be modulated by a variety of environmental and social contexts^39,40^, involving intrinsic physiological mediators (like stress hormones and neurotransmitters)^41–45^, or implicit processes (like priming)^46^. Most of these studies focused on the cognitive biases of populations with strong political positions, due to identification with specific political or ideological wings.

The aim of the present research is to evaluate the priming susceptibility on decisions. Our working hypothesis is that mere repetition of an option (repetition priming) or its emotional valence (emotional priming) would prime the choice preference, but this susceptibility to priming could be impaired by the task for which it is chosen (top-down modulation). By top-down modulation, we mean the influence of top-down factors such as attention, intention and—in particular, in our case—the task involved in the DM processes. We assume that the task representations carried out in the prefrontal cortex regulate the neural mechanisms underlying Type-1 and Type-2 processes. Consistent with our hypothesis, several papers have pointed out that memory processes can contribute to a broad class of decisions^47^, especially priming, a case of implicit memory^46^. Though, part of the published bibliography evaluating the priming effect on cognitive processes could not be replicated (i.e. subliminal priming), this could be explained by the conditions where priming could be effective, experimental designs, or bias during results’ analysis^48^. Prior repeated exposure to a stimulus was investigated to induce recognition and familiarity to it (repetition priming)^49–53^, and some social and political studies suggest an effect on political decisions^54–56^. On the other hand, an effect of emotional or affective priming was also described^57–62^. Facial expressions can be subtle cues on social decisions^60–63^. Inferences of competence from a face’s appearance (first impressions), suggesting rapid, unreflective trait inferences, can contribute to voting choices^64–66^, an effect observed even in children^67^. Furthermore, a retrospective study of U.S. presidential elections suggests that knowledge of biographical information about candidates can be a good predictor of results^68^.

Could social context variables prime complex decisions? Could top-down processes impair this priming susceptibility? These are the questions that guide the design of our cognitive experiments and the analysis of the data obtained during the 2019 Argentine presidential election (a social study). We aim to assess whether repeated exposure to certain visual stimuli (faces in the cognitive experiments or candidates in the social study) or association with emotional valence, can act as priming for complex DM processes. Understanding how information about candidates is cognitively used by voters to make a decision is an important question to capture the overall picture of political life. To address these questions, we tested our hypothesis in controlled cognitive experiments and contrasted the results with a big data analysis generated from variables obtained during the 2019 Argentine presidential election.

## Results

**Figure 1:**
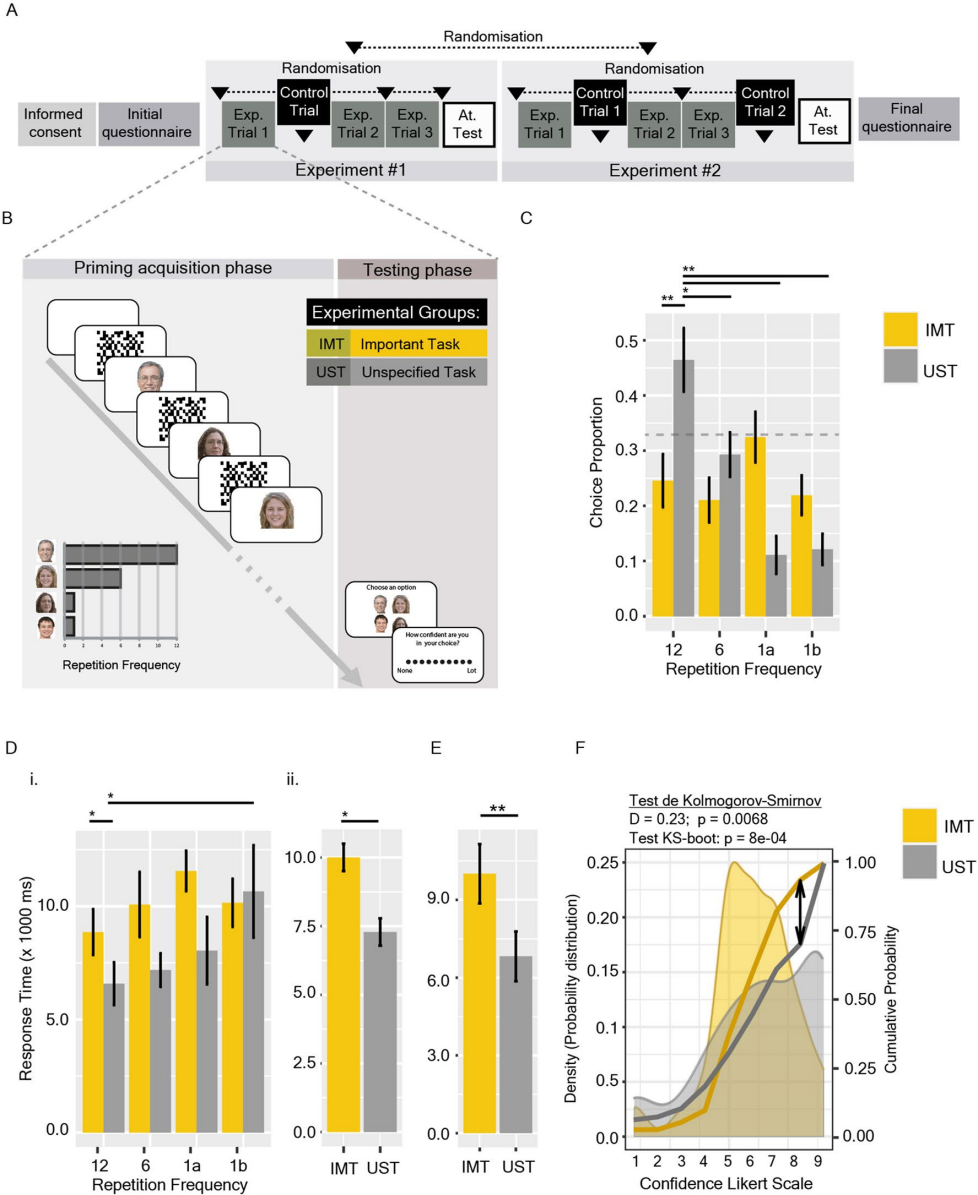
Repetition priming induces a choice preference associated with a lower response time in the Unspecified Task group but not in the Important Task group. (**A**) Experimental Series #1 design. The order in which the Experiment #1 and Experiment #2 appear, as well as the Control Trial position (Exp#1) and the Control Trial #1 or #2 (Exp#2), were all randomised for each participant. (**B**) Experimental design for Experiment #1: each experimental trial consists of two stages: a priming acquisition and a testing phase. The first one consists of faces (presented 12, 6 or 1 times; 200 ms face presentation) randomly presented, interspersed by a mask. The second one, in the election of only one face and the confidence of the choice. (**C**) Choice Preference as the proportion of the chosen face over a total of 3 trials. (**D**) Response Time separated by the chosen face frequency (i) or by group (ii). (**E**) Response Time, desegregated by group, of Control trials. (**F**) Probability Distribution of reported confidence. Areas show the density probability distribution; yellow and grey lines show the cumulative probability. Differences in the cumulative probabilities were analysed by the Kolmogorov–Smirnov (KS) Test: the arrow revealed the highest distance between distributions. Significances were also analysed by bootstrapping. Bars shown mean ± SEM. The grey dashed line represents the expected proportion for random choice (null hypothesis). *p < 0.05, **p < 0.01.

**Figure 2:**
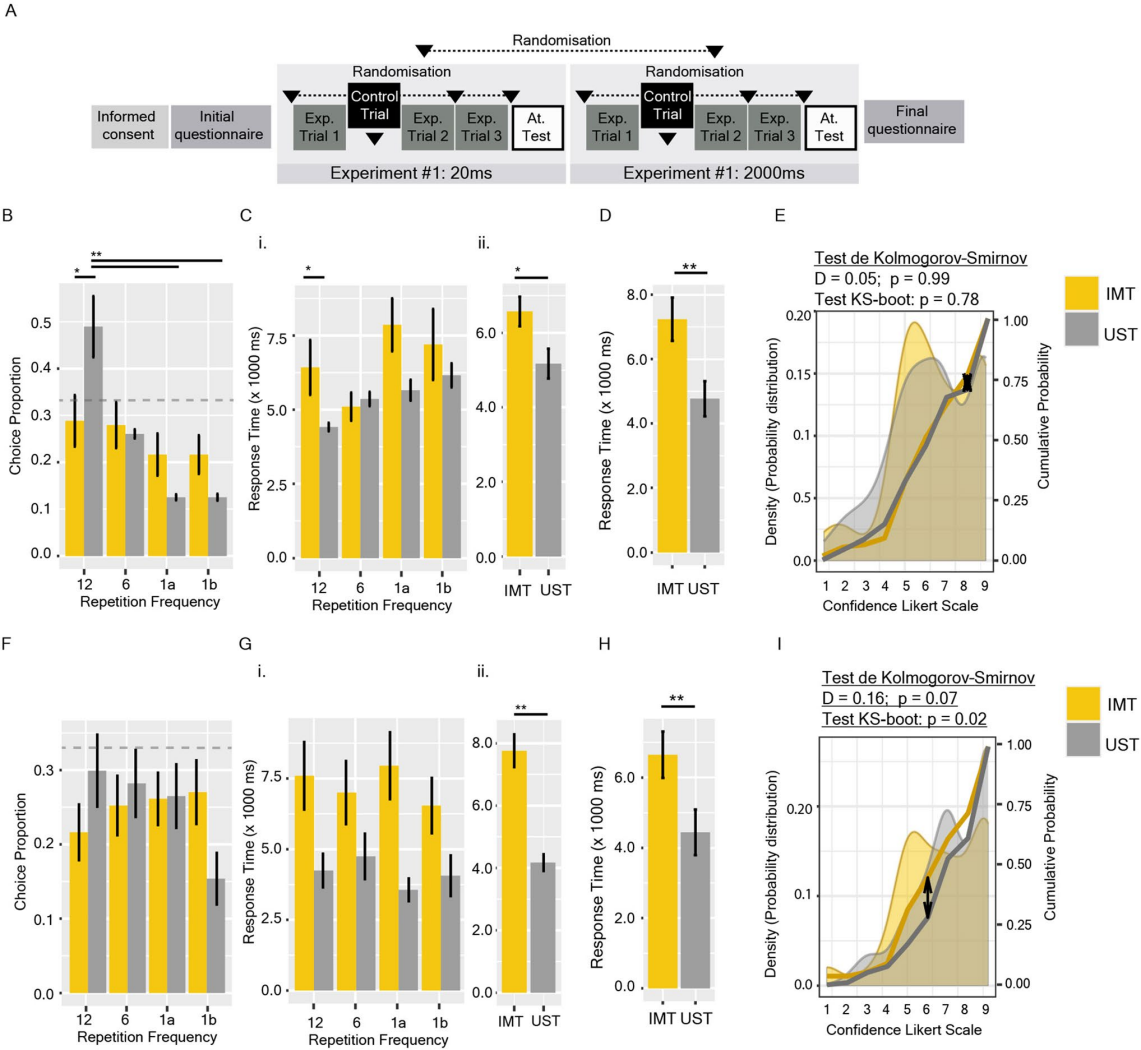
Parametric conditions under which repetition priming induces choice preference. (**A**) Experimental Series #2 design. The order in which both versions of Experiment #1 [Subliminal condition: 20 ms face presentation; Lasting condition: 2000 ms] appear, as well as Control Trial position, were all randomised for each participant. (**B**–**E**) Subliminal condition: (**B**) Choice Preference as the proportion of the chosen face over a total of 3 trials. (**C**) Response Time separated by the chosen face frequency (i) or by group (ii). (**D**) Response Time, desegregated by group, of Control trials. (**E**) Probability Distribution of reported confidence. (**F**–**I**) Lasting condition: (**F**) Choice Preference as the proportion of the chosen face over a total of 3 trials. (**G**) Response Time separated by the chosen face frequency (i) or by group (ii). (**H**) Response Time, desegregated by group, of Control trials. (**I**) Probability Distribution of reported confidence. Areas show the density probability distribution; yellow and grey lines show the cumulative probability. Differences in the cumulative probabilities were analysed by the Kolmogorov–Smirnov (KS) Test: the arrow revealed the highest distance between distributions. Significances were also analysed by bootstrapping. Bars shown mean ± SEM. The grey dashed line represents the expected proportion for random choice (null hypothesis). *p < 0.05, **p < 0.01.

For cognitive experiments, a Priming-Induced Decision-Making Paradigm [PIDM] was developed, consisting of choosing a face from four options, whose parametric characteristics were defined a priori but randomly assigned to each face (“Methods”). All cognitive experiments were conducted online due to the COVID-19 pandemic during 2020–2021, using our open access platform: https://experimentoscognitivos.com. A total of 242 adults (137 women; Supp. Table 1) participated in the cognitive experiments, randomly assigned to two experimental groups: the first one was asked to choose a face for an important task [IMT group; instructed by the question “Who do you choose for an important task?”]; while the second group was asked to choose a face with unspecified task [UST group; instructed by the question “Who do you choose?”]. Three Experimental Series were performed. The first consisted of a random concatenation of Experiment #1 and Experiment #2 (Fig. 1A), while Experimental Series #2 consisted of a random concatenation of two versions of Experiment #1 (Fig. 2A; “Methods”). The third Experimental Series consisted of a concatenation of Experiment #1 and Experiment #2, but in this case, it was synchronic and the choice preference was tested again 24 h after priming session.

On the other hand, a total of 3673 adults (1845 women; Supp. Table 1) participated in the surveys developed to assess several social and subjective variables during the 2019 Argentine presidential elections (“Methods”). Two election periods were analysed through the surveys: Period #1 [from 23-06-2019 to 08–08-2019] and Period #2 [from 01-09-2019 to 24-10-2019]. During Period #2, 22,510 newspaper articles were collected from the main national print media (Suppl. Table 4) to evaluate the frequency of mention of the different presidential formulas/candidates and their emotional associations.

All participants excluded for not meeting any inclusion criteria and the reasons are detailed in Supplemental Table 1.

### Repetition priming induces a choice preference only in the Unspecified Task group

Could mere repetition of a stimulus promote its choice? To address this question, in Experiment #1, the paradigm was applied in such a way that 4 faces were shown in a random order repeatedly. One face was shown 12 times, another one 6 times, and the remaining two only 1 time each during the acquisition phase (Fig. 1B). Then, participants were asked to choose one of these (4) faces for an important task [IMT group] or without any specification [UST group], and for the confidence of their choice. This sequence was repeated 3 times for each participant as independent experimental trials (with different faces for each one), but randomly interspersed with a control trial in which the 4 faces were shown 5 times each (Fig. 1A). Deviance analysis of the Multinomial Logit Model showed a significant group effect [LR Chisq = 23.63, p = 2.98e−05]. Participants in the UST group chose the faces that were shown 12 times significantly more, while those in the IMT group were more likely to choose faces presented once (Fig. 1C). Response Time (RT), analysed by Generalised Linear Models, in the experimental trials was significantly lower in the UST group than in the IMT one [Chisq = 5.55, p = 0.018] (Fig. 1D), but also in control trials [Chisq = 5.14, p = 0.023] (Fig. 1E). Reported confidence showed significant differences in-between groups when analysed as cumulative distribution (Fig. 1F), suggesting lower confidence in the IMT group. An extended analysis is shown in Extended Results.

To evaluate the parametric conditions under which this priming is effective, Experimental Series #2 was designed (Fig. 2A). In Experiment #1 of Experimental Series #1 (above), each face presentation lasted 200 ms (enough time to consciously perceive each face^69^), followed by a mask for another 200 ms. In Experimental Series #2, subjects performed two versions of the original experiment: in one version, the presentation time of each face was 20 ms [the subliminal condition], while in the other, it was 2000 ms [the lasting condition] (“Methods”). Consistent with Experiment #1’s results, under the subliminal condition, participants in the UST group chose the faces repeated 12 times significantly more than those in the IMT group [LR Chisq = 10.68, p = 0.013] (Fig. 2B). Under this condition, RT of the UST group was also significantly lower than for the IMT group [Chisq = 4.78, p = 0.028] (Fig. 2C,D), but confidence did not show significant differences between groups (Fig. 2E). Interestingly, under the lasting condition, differences in choice preference were not more significant in between groups [LR Chisq = 4.96, p = 0.17] (Fig. 2F). However, RT shown higher differences when groups were compared [Chisq = 19.97, p = 7.83e−6] (Fig. 2G,H), and confidence was also lower in the IMT group (Fig. 2I).

### Emotional semantic priming induces a strong choice preference

**Figure 3:**
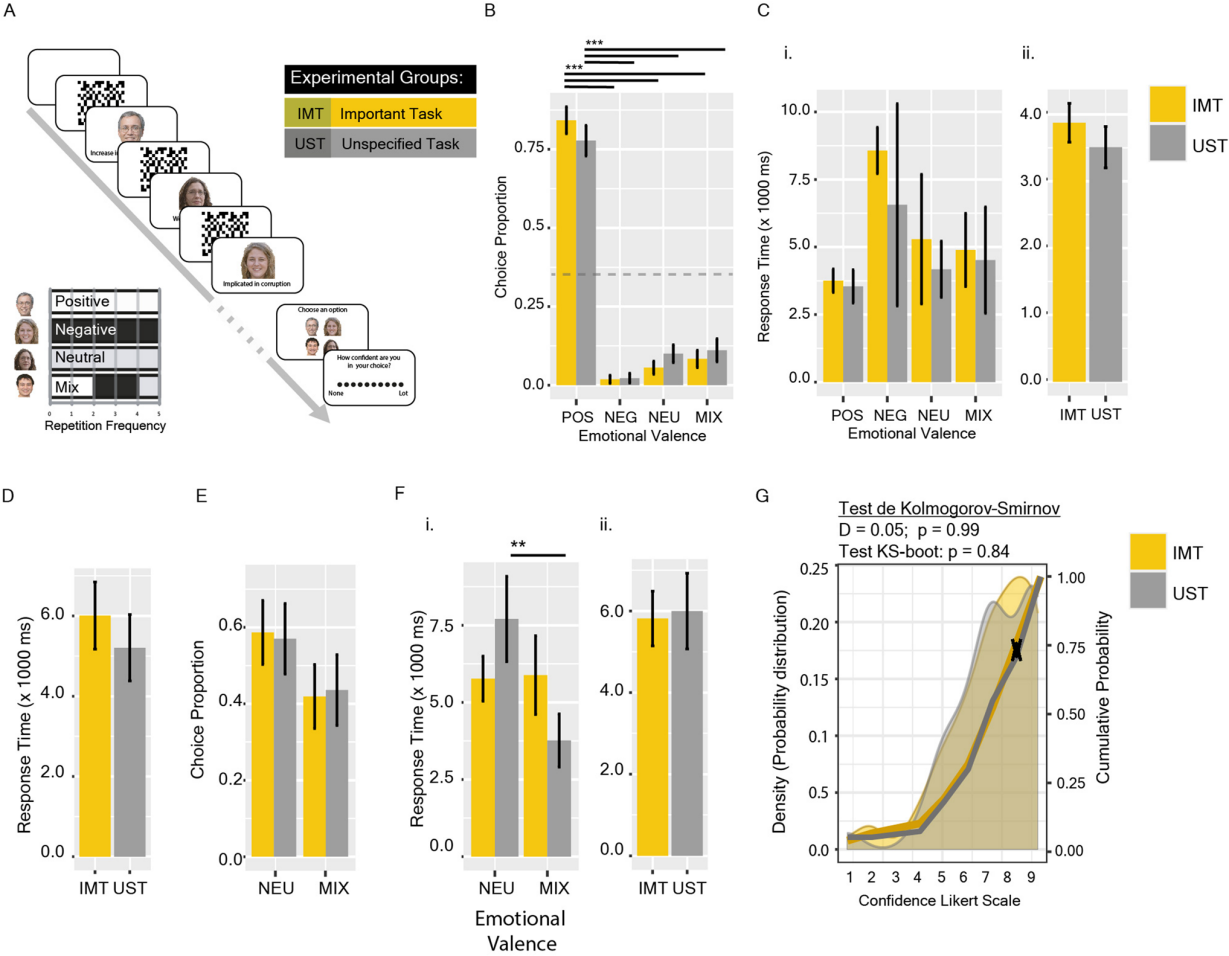
Positive emotional semantic priming strongly induces a choice preference, independent of the experimental group. (**A**) Experimental design for Experiment #2. (**B**) Choice Preference as the proportion of the chosen face over a total of 3 trials. (**C**) Response time separated by chosen emotional valence face (i) or by group (ii). (**D**) Response time of Control #1. (**E**) Choice Preference in Control #2. (**F**) Response time of Control #2, separated by chosen emotional valence face (i) or by group (ii). (**G**) Probability Distribution of reported confidence. Areas show the density probability distribution; yellow and grey lines show the cumulative probability. Differences in the cumulative probabilities were analysed by the Kolmogorov–Smirnov (KS) Test: the arrow revealed the highest distance between distributions. Bars shown mean ± SEM. The grey dashed line represents the expected proportion for random choice (null hypothesis). **p < 0.01, ***p < 0.001.

Occasionally, in political elections, we have to make a choice between candidates equally familiar to us. In these cases, could the emotional valence of the information we access about them influence choice? Experiment #2 (from Experimental Series #1; Fig. 1A) addresses this question. Here, the paradigm was adapted so that all faces were shown only 5 times, each in a random order, but in this case, they were presented simultaneously with phrases. Faces were assigned randomly to four different categories, depending on the emotional valence of the phrases: positive (5 phrases), negative (5), neutral (5) or a mix of positive (2), negative (2) and neutral (1) phrases (Fig. 3A). Phrases were previously created under certain grammatical criteria, with semantic political content (or not for neutral phrases), and rated by 72 participants through a 5-point Likert-like scale from very unpleasant to very pleasant (Suppl. Table 2; “Methods”).

Similar to Experiment #1, after the acquisition phase, participants were randomly assigned to UST or IMT groups. The complete experimental sequence involved 3 trials (with different faces and phrases for each one), randomly interspersed with 2 different control trials. In Control #1, the 4 faces shown 5 times were associated in all cases with neutral phrases, while in Control #2, two faces were associated with neutral phrases and the other two with the mixed combination.

Participants from both groups strongly preferred to choose the face associated with positive phrases [LR Chisq = 12.26, p = 0.006] (Fig. 3B). However, no significant differences were observed between groups in choice preference [LR Chisq = 2.02, p = 0.56] (Fig. 3B), response time [Chisq = 2.43, p = 0.11] (Fig. 3C,D), and confidence (Fig. 3G). These results suggest that the emotional valence of the phrases can strongly prime the decision process, regardless of the nature of the task. Nevertheless, due to the experimental design, it is not possible to determine whether the positive or negative valence has equivalent but reversed priming power, or different. To discern this, Control #2 allowed us to analyse the impact of negative or positive phrases (in the mixed category faces) in comparison with neutral content. No differences were observed (Fig. 3E,F; Suppl. Material).

### Long-term effect of the repetition and emotional semantic priming

**Figure 4:**
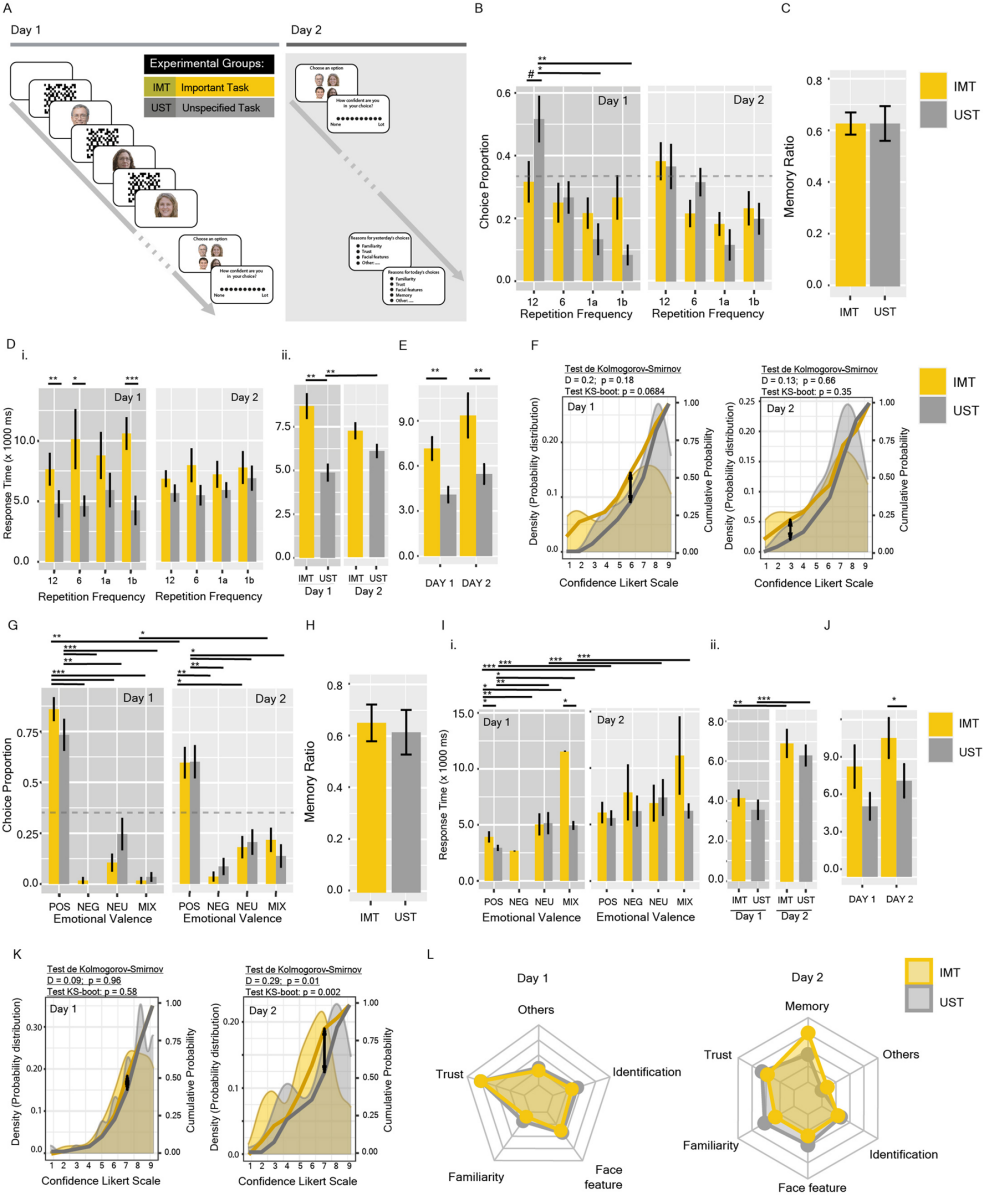
Long-term effects of repetition and emotional semantic primings. (**A**) Experimental design for Synchronic Experiment #1. (**B**–**F**) Synchronic Experiment #1: (**B**) Choice Preference (as the proportion of the chosen face over a total of 3 trials) at day 1 and day 2. (**C**) Memory Ratio, calculated as the number of faces that were chosen again on day 2 over the total number of choices. (**D**) Response time separated by chosen frequency (i) or group (ii). (**E**) Response time for Control #1. (**F**) Probability Distribution of reported confidence. Areas show the density probability distribution; yellow and grey lines show the cumulative probability. Differences in the cumulative probabilities were analysed by the Kolmogorov–Smirnov (KS) Test: the arrow revealed the highest distance between distributions. (**G**–**K**) Synchronic Experiment #2: (**G**) Choice Preference (as the proportion of the chosen face over a total of 3 trials) at day 1 and day 2. (**H**) Memory Ratio, calculated as the number of faces that were chosen again on day 2 over the total number of choices. (**I**) Response time separated by chosen emotional valence (i) or group (ii). (**J**) Response time for Control #1. (**K**) Probability Distribution of reported confidence. (**L**) Stated reasons for the choices of day 1 and day 2. Spider chart shows frequency of selection of each reason. Bars shown mean ± SEM. The grey dashed line represents the expected proportion for random choice (null hypothesis). ^#^p = 0.0567, *p < 0.05, **p < 0.01, ***p < 0.001.

Could these two types of priming persist in the long-term? To evaluate the persistence of the priming effect, we conducted Experimental Series #3. This consisted of a two-session synchronic experiment, online, but monitored by the researcher via video meeting. On the first day, participants completed the acquisition and test sessions, consisting of a randomised sequence of Experiment #1 and Experiment #2. On the new day, 24 h later, they completed new test sessions. Afterwards, they were asked about the reasons for their choices (Fig. 4A; “Methods”).

The synchronic experiment #1 (for day 1) replicated the previous results observed under uncontrolled online (asynchronic) conditions. Although deviance analysis of the MLM did not show a significant group effect [LR Chisq = 6.30, p = 0.056], post hoc analysis showed significant differences between frequencies for the UST group, but not for the IMT (Fig. 4B). No significant differences were observed between groups or frequencies for day 2 (Fig. 4B). With respect to Response Time, a significant effect was also observed for GROUP variable on the first day [Chisq = 10.28, p = 0.001], but not on the second day [Chisq = 2.86, p = 0.09] (Fig. 4D,E). Confidence did not show significant differences at day 1, although the trend is in accordance with the previous result (Fig. 4F). Analysis of the Memory Ratio [estimated as the number of faces that were chosen again on day 2 over the total number of choices] did not show any significant effect associated with GROUP or the FREQ chosen on the 1st or 2nd day (Fig. 4C).

On the other hand, in synchronic Experiment #2, although the differences in the preference of faces with different emotional valence flattened on day 2, they were still significant. However, the IMT group (but not UST) showed a significant decrease in the choice preference of positive faces (Fig. 4G). Unlike the asynchronous experiment, here we observed in-between groups differences in RT at day 1 [Chisq = 6.57, p = 0.01], but not at day 2 [Chisq = 0.37, p = 0.53] (Fig. 4I,J). In addition, analysis of reported confidence at day 2 showed that the IMT group had a significant lower confidence than UST (Fig. 4K). Analysis of the Memory Ratio did not show a significant effect associated with GROUP but to the valence of the face chosen the 2nd day [LR Chisq = 60.16, p = 5.43e−13] (Fig. 4H). When participants were asked retrospectively about the reasons for their choice on day 1, no differences between groups were observed, with "Trust" prominent in both. However, on day 2, “Memory” was a more prominent reason in the IMT group while “familiarity” was given more by the UST group (Fig. 4L).

### A social study: 2019 Argentine Presidential Election

Up to now, these cognitive experiments support the idea that the repetition or emotional valence associated with a stimulus (a face) could be effective in inducing its choice. Could these cognitive mechanisms occur during election campaigns in a way that promotes voting for particular candidates? To approach an answer, we have taken advantage of the 2019 Argentine Presidential Election to assess subjective and social variables (Suppl. Fig. 1; “Methods”). The Argentine Presidential Election consists of two stages. The first, named PASO [August 11th, 2019], is intended to filter the main presidential candidates that will be present in the General Election [October 27th, 2019]: only candidates who obtain more than 1.5% of the votes in the PASO advance to the General Elections. Candidates who reached this goal were M. Macri [MM], A. Fernandez [AF], R. Lavagna [RL], N. Del Caño [NDC], J. Gomez Centurion [JGC], and J.L. Espert [JLE] (Suppl. Table 6).

We hypothesised that greater exposure to the image or information about the candidate favours greater familiarity, just as greater association with positive emotional valence would favour greater trust. In turn, greater familiarity with and/or greater trust in candidates would favour their election. To test our hypothesis, we conducted online surveys from June 22nd to August 11th [Period #1] and from August 12th to October 27th [Period #2], to estimate Familiarity, Trust and Voting Probability for each candidate, and the used social and press media to get information about candidates (Suppl. Table 3). During Period #2, newspaper articles were collected from news media websites as a sub-sample of the candidate’s exposure to estimate the number of mentions of each candidate and the positive or negative perception of them, as associated with the headlines.

**Figure 5:**
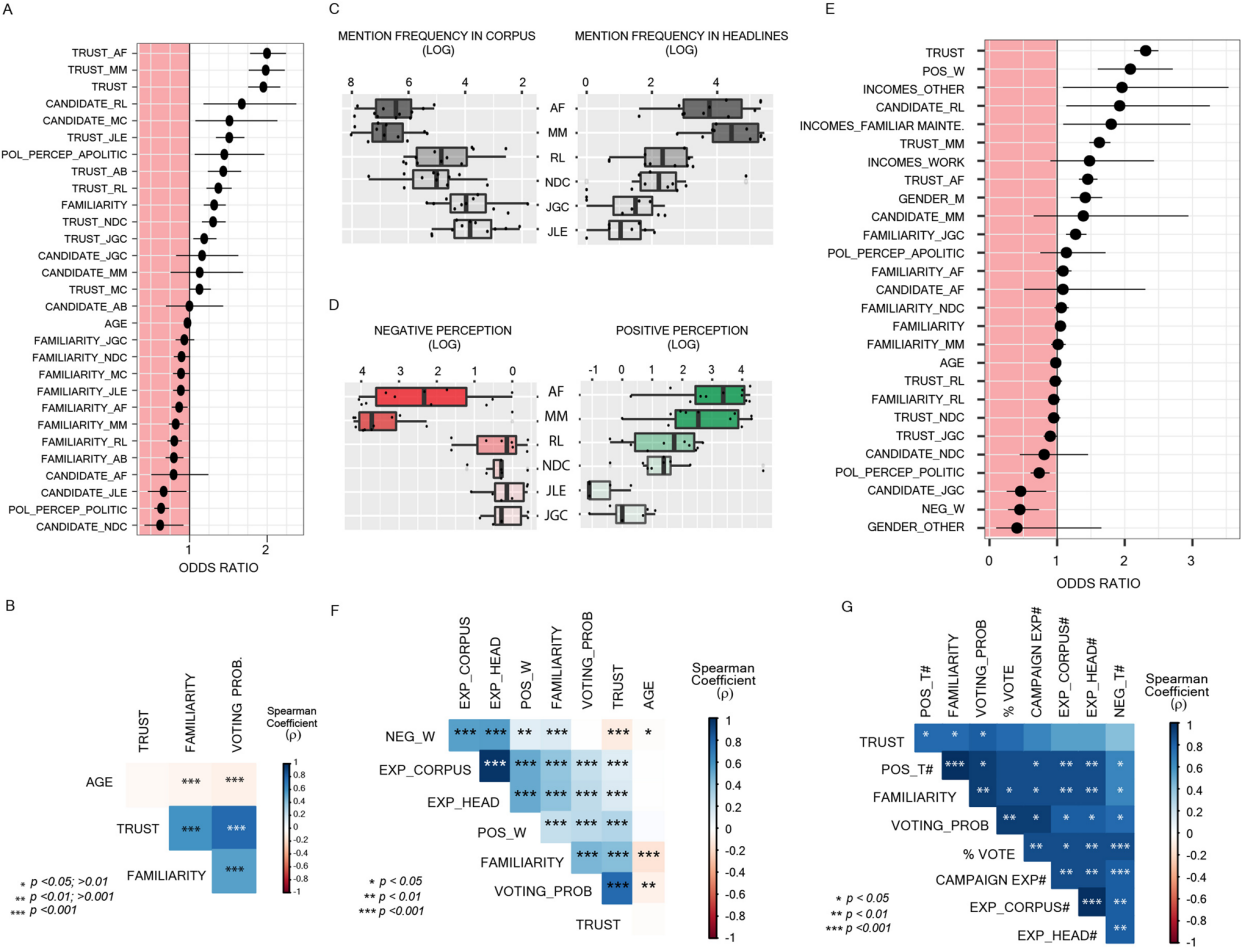
Social Study: 2019 Argentine President General Election. (**A**) Odds ratios ± confidence intervals of the final model with only the significant variables for Period #1. (**B**) Spearman correlation matrix of variables for each candidate (Period #1). (**C**) Frequency of each candidate’s mention in either the corpus or the title of the newspaper articles published between September 21 and October 27, 2019, in the main written media. Each dot corresponds to each medium’s candidate mention. The presented order of candidates corresponds to the order of the election results; the intensity of the grey filler corresponds to the number of obtained votes. (**D**) Sentiment analysis of the news’ headlines that mentioned at least one candidate or political force. Figure shows positive or negative targets assessed as subjects’ perception. Each dot corresponds to each medium’s candidate positive or negative target. The presented order of candidates corresponds to the order of the election results; the intensity of the red (negative) or green (positive) filler corresponds to the number of obtained votes. (**E**) Odds ratios ± confidence intervals of the significant variables of the ordinal model. (**F**) Spearman Correlation matrix of variables for each candidate. (**G**) Spearman Correlation matrix of the means of each variable for each candidate and the Campaign Expenses and Election results as the percentage of vote. *AF* Alberto Fernandez, *MM* Mauricio Macri, *RL* Roberto Lavagna, *NDC* Nicolas Del Caño, *JGC* Jorge Gomez Centurion, *JLE* Jose Luis Espert, *POS_T and NEG_T* total positive or negative mentions in the headlines of news articles for each candidate, *POS_W and NEG_W* weighted positive and negative perception of each participant according to the media they use to get candidate information, *POL_PERCEP* political self-perception, as POLITICAL or APOLITICAL person, *GENDER_M* (male), *EXP_CORPUS or EXP_HEAD* weighted mention in news corpus or headlines according to the media that each participant used to get information about candidate. *VOTING_PROB*. voting probability, *% VOTE* percentage of votes for each candidate during General Election, *CAMPAIGN_EXP* declared campaign expenses of each candidate. ^#^Expressed as LOG. *p < 0.05, **p < 0.01, ***p < 0.001.

Analysing data with a Multinomial Ordinal Model, we observed for Period #1 that Trust and Familiarity mostly explain the variability of Voting Probability for each candidate [Odds Ratios (CI 2.5–9.75%): Trust: 1.95 (1.75–2.16); Familiarity: 1.31 (1.18–1.46)] (Fig. 5A; Extended Results). Voting Probability correlated more with Trust [Spearman coeff. (ρ) = 0.82] than Familiarity [ρ = 0.56], though both were significant (p < 0.001) (Fig. 5B). Interestingly, for Period #2, the Odds Ratios (OR) for Trust increased [2.3 (2.13–2.49)] and for Familiarity decreased [1.04 (0.97–1.13)] (Fig. 5E), compared to Period #1. These results suggest that Familiarity may have a greater impact on the PASO than on the General Election, and Trust a greater impact on the General Election.

During Period #2, the analysed news articles revealed an asymmetry in the mentions of each candidate (Fig. 5C) as well as in the positive or negative perception of their headlines (Fig. 5D). Weighted positive and negative perceptions showed a great impact on Voting Probability [Odds Ratios (CI 2.5–9.75%): POS_W: 2.08 (1.60–2.70); NEG_W: 0.44 (0.27–0.73)] (Fig. 5E). Weighted mentions of candidates in the news corpus or in the headlines (EXP_CORPUS or EXP_HEAD) were not significant variables, so they were not included in the final model. However, when the relationship between variables was analysed using the Spearman correlation, both variables were significantly associated with Familiarity [EXP_CORPUS: ρ = 0.39; EXP_HEAD: ρ = 0.40; p < 0.001]; Trust positively correlated with POS_W [ρ = 0.33; p < 0.001] but negatively with NEG_W [ρ = − 0.15; p < 0.001]; and Voting Probability correlated better with Trust [ρ = 0.81; p < 0.001] than Familiarity [ρ = 0.47; p < 0.001] (Fig. 5F).

For each subjective variable, the mean per candidate was calculated and a cross-correlation analysis was performed to evaluate their relationship with campaign expenses (CAMPAIGN_EXP) and electoral results. The percentage of votes shows a significant correlation with Voting Probability [ρ = 0.94; p = 0.005], Familiarity [ρ = 0.87; p = 0.022], and campaign expenses [ρ = 0.95; p = 0.003] (Fig. 5G).

## Discussion

**Figure 6:**
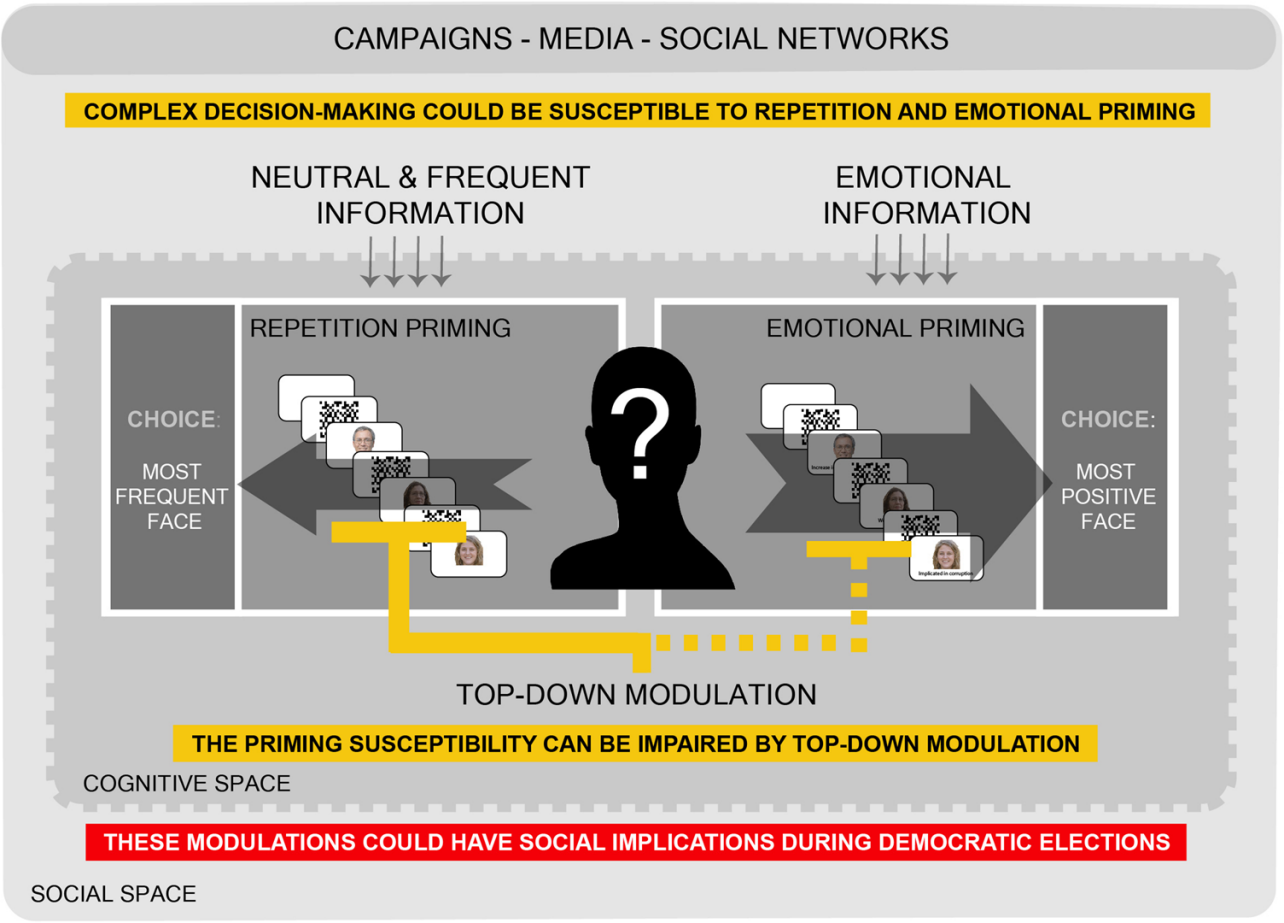
Integrative scheme of priming susceptibility and top-down modulation on Complex Decision-Making processes, at the cognitive and social level. Complex decision-making processes may be susceptible to repetitive and emotional priming, inducible by repetitive exposure to neutral or emotionally charged information (through campaigns, social networks or media). This susceptibility may be affected by top-down modulation. These processes may have social implications at the level of electoral processes.

Can the mere repetition of a stimulus or its association with a positive emotional valence induce its choice? Can this cognitive mechanism be used to induce the choice of a candidate? Underlying these questions lies the alleged susceptibility of complex decision-making (DM) processes to intentional social manipulation. In the present study, we observed that repeated exposure to a face or its association with a positive valence could prime the choice of a face, and at least repetition priming could have effects even in conditions of unconsciousness. Moreover, the temporal dynamics and strength of these priming are equivalent to those observed for other mnesic processes. Last but not least, this priming could be impaired by top-down modulation, like the importance of the task for which it is chosen (Fig. 6). Several social and political science studies have previously suggested that prolonged exposure to election campaign content or association with emotional content may favour the election of a candidate^54–56^. In this way, unequal access to campaign funding or political alliances with the media can have an asymmetrical impact on familiarity or trust towards target political forces. If this is the case, democracy could easily be reduced to a matter of marketing.

To evaluate the susceptibility to priming of complex decisions, and their top-down modulation (triggered by instructions), we developed an experimental paradigm that allowed us to assess these effects by online experiments based on the choice of a single face over multiple options. We consider this a noteworthy factor since most political choices are made over multiple options, although choice strategies may include categorising options into two categories (eligible or ineligible)^19^. In all experiments, participants were randomly assigned to either of two experimental groups, defined by the instructions, in order to evaluate whether top-down modulation could alter susceptibility to priming. In IMT group, participants were asked to make their choice for an important task, while in the UST, to make it without any specification. In our paradigm, while all participants knew a priori that the research project was framed by complex decisions such as political choice, the instruction in the IMT triggers a more canonical DM process mediated by potential benefit. However, the UST group was originally conceived as a control group, where the decision is made without any instructed motivation or under uncertainty. We hypothesised that, under these conditions, the decision-makers are more susceptible to social modulation, done here by repetition priming and emotional priming. Motivation^70,71^ and mood^72^ can both affect the way people make judgements about options, reasons and decisions. From a political perspective, these psychophysiological states could be induced by political campaigns based on negative content (affairs, fake news, etc.), which generate an increase in key variables during the political DM process (disgust, cynicism, efficacy, apathy, unwillingness to vote, distrust, etc.), although social and political literature is not conclusive in this regard^73–75^. However, theories about emotions offer useful frameworks for evaluating this effect from a cognitive perspective^75^. One of the goals of this work is to interpret our results from an interdisciplinary perspective that integrates both levels of complexity (cognitive and social).

In order to analyse the behavioural differences in both experimental groups, Dual Process Theories (DPT) provide a relevant theoretical framework. They propose two different types of processes involved in reasoning and DM, either in a dichotomous^11,15–17^ or a hybrid continuous way^16,18,19^. Type-1 processes were characterised as fast and automatic and to involve (context dependent) implicit processing occurring incidentally and without concomitant awareness; whereas Type-2 processes involve explicit, deliberate, flexible and slow processing, always accompanied by awareness^16,76,77^. While most of these DPT consider interaction between the two types of processes, alternative hybrid models suggest a continuum between both, and even distinguish between processes based on implicit learning (Type-1) and automatic processes, defined by an individual’s ability to consciously access behaviour but not the ability to control it^16^. From the DPT perspective, the DM process involved in the UST could be well explained by Type-1 or automatic processes, while in IMT, the instructions could induce Type-2 processes^19^. Our experimental paradigm allowed us to use the instructions to induce both types of processes in DM and distinguish both by their susceptibility to priming and response times.

Experiment #1 and Experiment # 2 were designed to evaluate the susceptibility to repetition priming or emotional semantic priming, respectively, in the DM process of each experimental group. The main reason we evaluated the repetition priming traces back to the potential impact of repeated exposure to visual content during election campaigns^56^, favouring perception of familiarity. Otherwise, the perception of trust or distrust is induced when a face is associated with positive or negative content^78^, respectively.

When each face presentation lasted 200 ms [Experiment #1 from Experimental Series #1], we observed that the UST group mostly chose the faces that were shown 12 times, taking significantly less time to choose and with higher confidence than the IMT group. Results under the subliminal condition were consistent with the first experiment. However, the lasting condition did not show preference of choice for faces that were shown 12 times, but the response time remained longer in IMT, suggesting that priming and top-down modulations are independent processes. Why did the UST show no susceptibility to priming under this condition? One possible explanation could be that the minimum time threshold for a stimulus (either single or cumulative) to prime the decision is less than 2000 ms, so that in this condition all four categories’ faces reach the threshold equally before the testing phase (saturation hypothesis). A second hypothesis is that, since the acquisition phase is so long, choice processes are already taking place in this phase. Thus, in this condition, different processes could be overlapping, so that the UST’s participants arrive at the choice having carried out more unintentional reflexive processes (overlapping hypothesis). Overall, the UST group chose the most repeated faces, faster and with a higher confidence than the IMT group, a metacognitive variable that indirectly reflects the fluency of the cognitive process, suggesting a Type-1-like process. In contrast, in the IMT group the decision seems to be equivalent to a Type-2-like process: participants showed no preference in relation to the frequency of repetition of the stimulus, took longer in the decision making process, and made the decision with less confidence, suggesting conflicting setbacks^16^.

On the other hand, Experiment #2’s results showed that both groups predominantly chose faces associated with positive valence, suggesting that the task instruction here does not induce any differential processing. One possible explanation could be that this emotional priming, with semantic political content, induced in both groups the feeling that they must choose the person for an important task (whether or not it was explicit in the instruction). However, response time was low for both groups, compared to the ones previously observed in Experiments #1 for the UST. A plausible explanation is that, similar to what we speculate occurred in the lasting condition of Experiment #1 (where the faces were also presented for 2000 ms), the decision process could begin during the acquisition phase. However, in contrast to that experiment, no differences in response times between groups were observed in Experiment #2. Finally, it may be that, under the specific parametric conditions of our experimental design, priming is so strong that no top-down modulation is enough to induce a more reflective decision process. As we will discuss below, this could explain the result better.

Since our original question was whether these cognitive mechanisms can play a role over time (during election campaign processes) in favouring the election of certain candidates, we evaluated the long-term effects of such primings. In Experimental Series #3, synchronic experiments were conducted in two sessions separated by 24 h. On the first day, experiments were equivalent to those in Experimental Series #1, except for the fact that participants conducted them in a synchronic manner. Previous results were reproduced here under these more monitored experimental conditions. On day 2, the participants only had to choose from the faces seen the day before without any priming applied during this day. Here, however, the results were different. For Experiment #1, differences between groups in the choice preference, response time or confidence were not detected anymore. For Experiment #2, we observed a decrease in the preference for positive faces (especially in the IMT), but not any significant differences in choice preference, response times and confidence between groups. However, while response times from both groups were not significantly different in control #1 (without priming) at day 1, they were at day 2. In addition, no differences were observed in memory ratio, for both experiments. However, when analysing the significant effects, we observed that the valence of the face chosen the 2nd day could explain the memory ratio for Experiment #2 (suggesting that the priming could be operating during evocation processes). The reasons participants reported for their choices did not show differences between the groups for day 1, but for day 2, “memory” was the most predominant reason in the IMT group, suggesting that although the memory ratio was not different from the UST group, they were more aware of the reasons for their choices.

Another fundamental aspect to be discussed is that these primings seem to present similar dynamics to other mnesic processes, such as forgetting. Although our experimental design does not allow us to assess whether priming enters into consolidation, the Endogenous Modulation of Memory Consolidation Theory provides a theoretical framework for interpreting our results. During consolidation, neuromodulatory systems (mediated by hormones and neuropeptides), triggered by emotional experiences, can modulate the strength of the memory; that is, how well the memory would be evoked^79^. From this point of view, emotional priming could be stronger because it involves an emotional memory, but 24 h later, priming strength decayed, mainly in the IMT group. Top-down information could have been integrated into priming memory during acquisition and/or be a key cue during recall, which would explain the differences between the groups on day 2. On the other hand, repetition priming did not seem to be as strong. Further experiments are necessary to establish whether a greater number of face repetitions, or reinforcement over time, is a sufficient condition for this priming to be expressed in the long-term. For a more ecological interpretation of the phenomenon, we can assume that exposure to visual or emotional content of the candidates occurs continuously throughout an electoral campaign, favouring a continuous perception of familiarity in the first case, and trust in the second.

These aspects were specifically analysed in relation to the social study. Numerous studies have assessed the relationship between subjective variables (such as trust) in political DM processes from the perspective of political psychology and social political sciences^54–56,78,80^. Some have analysed the role of social media, print media, campaigns and fake news in the formation of judgement criteria and political decisions^35–38,73,74,80–82^. Unconscious detection of self-deception was detected in populations of pro- versus anti-government voters during the 2015 presidential elections in Argentina^30^ and Sweden^83^. Social cognition allows us to establish bridges between experimental cognitive studies and those equivalent at a social level. The aim of the social study was precisely to challenge our hypotheses tested in the previous cognitive experiments at a higher level of complexity and by means of a more ecological DM task: the election of a president.

Of all the explanatory variables analysed, trust and familiarity are the ones that best explain the variability of the voting probability for each candidate. But how is this trust or familiarity induced? As detailed above, our hypothesis is that the frequency of recurrent exposure to the image or information about the candidate favours their familiarity. To test our hypothesis, we analysed the frequency of mentions of each candidate in the mainstream media, as an operationally accessible sub-sample of such exposure. Another indicator could be the declared campaign expenses of each candidate. And indeed, both variables correlated very well with the familiarity of the different candidates. The other challenged hypothesis was that the association with positive valence in the mentions could influence the perceived trust for each candidate. A sentiment analysis was performed on news headlines associated with candidates, which allowed us to estimate indicators of positivity or negativity associated with them. Both indicators correlated very well with trust.

These approaches have several limitations. First, voters' actual exposure to neutral or emotionally charged images or mentions of candidates was under-sampled, although it allows for an indirect assessment of these parameters at the social level, where voters develop their political lives. Secondly, it does not directly assess how each candidate is perceived, but indirectly through familiarity and trust, two variables subject to a certain degree of interpretation by the participants. Finally, the performed analysis does not allow us to conclude causal relationships between variables in political DM processes. However, analysed together with the cognitive experiments above, they do allow us to provide strong support for our hypotheses, both at the cognitive and social levels.

## Methods

To test our working hypothesis, online cognitive experiments and a social study were conducted. The cognitive experiments involved a task to be performed on an internet-connected device, employing a new decision-making (DM) paradigm developed and standardised for our purposes: Priming-Induced Decision-Making Paradigm [PIDM]. In turn, the social study consisted of the analysis of data obtained from online surveys and news articles from websites published during the 2019 presidential election in Argentina. The cognitive experiments allowed us to test if whether repetition or emotional semantic priming could influence complex DM processes; while the social study allowed us to analyse the correlations of candidates’ mentions in the news and their emotional association with Familiarity, Trust, Voting Probability, Campaign Expenses, and Election Results. Finally, the integrated analysis allowed us to challenge our hypothesis at a higher level of complexity: the social one.

### Participants

A total of 242 (137 women, 113 men, 2 other gender) native Spanish speakers and healthy volunteers participated in the cognitive experiments. For the social study, surveys were completed by 3673 participants (1845 women, 1800 men, 28 other gender) from all provinces of Argentina. Populations details and excluded subjects from the data analysis for not meeting the inclusion criteria are described in the Supplemental Table 1. All participants gave informed consent to participate in experiments or surveys, and for the publication of the obtained information, in agreement with the ethics committee of the Clinic Hospital “José de San Martín”, University of Buenos Aires.

### Priming-induced decision-making paradigm (PIDM)

Two versions of the PIDM paradigm were developed in order to evaluate the Repetition priming (Experiment #1) and the Emotional Semantic priming (Experiment #2) effects on DM. In this paradigm, participants were instructed to observe a series of faces and then choose one of them. Immediately after, they were asked to indicate the confidence of their choice on a Likert scale of 1 to 9, where 1 was not at all confident and 9 was very confident. Participants carried out 3 experimental trials, each one consisting of an Acquisition Phase (faces presentation) and the Testing Phase (asking for the choice preference and confidence) (Fig.1B). One or two control trials (depending on the experiment) were randomly inserted in between. After this, a recognition test was performed to monitor attentional performance during experiments (Attentional Test) (Fig.1A). All cognitive experiments were conducted on a digital platform, programmed in JavaScript: https://experimentoscognitivos.com. The faces used in the experiments were created digitally using the "This Person Does Not Exist" website [https://thispersondoesnotexist.com/]. The selection of the faces was done in order to avoid any bias in terms of binary gender, apparent age and complexion. The experiments were made to select for each trial 4 random faces from a dataset of 52, and to avoid using the same face again during the next trials of the experiment for each participant. The face randomisation allowed the minimization of the effect of the face features on the posterior elections, so that they do not influence the population results.

Before the experiments, participants were instructed to answer an Initial Questionnaire, and once finished, the Final Questionnaire. The obtained information gave a personal characterization of the participants as well as defined whether they reached the Inclusion Criteria or not.

### Experimental groups

To assess whether the top-down modulation of the experimental instruction could condition choice or the effect of priming on it, participants were randomly assigned to two experimental groups: (1) Important Task [IMT]: the choice is instructed by the question “*Who do you choose for an important task?*”; (2) Unspecified Task [UST]: the choice is instructed by the question “*Who do you choose?*”.

### Experimental series #1

In the first Experimental Series, Experiment #1 and Experiment #2 were run concatenated in random order (Fig. 1A). In this series, the face presentation time in Experiment #1 was 200 ms, and in Experiment #2 it was 2000 ms, as described below in the experimental designs of both experiments.

### Experiment #1: repetition priming

In Experiment #1, the repetition priming effect over DM was evaluated. For this purpose, in this version of the PIDM, each experimental trial consisted of the presentation of 4 faces with different frequencies: one face appeared 12 times, another 6 times and 2 faces only once, giving a total of 20 presentations (Fig. 1B). Each face presentation was either for 200 ms (for Experimental Series #1) or 20 or 2000 ms (for Experimental Series #2), interspersed by a 200 ms mask. The masks were generated randomly from face to face. Participants performed 3 experimental trials and 1 control trial, in which the 4 faces appeared randomly 5 times each. The faces, their order, and the trials’ order were all randomised for each participant. The platform was programmed in order to record: (1) the chosen face; (2) the frequency of the chosen face (Choice Preference); (3) its position in the randomly generated order; (4) the response time of the choice; (5) the confidence; and (6) the number of faces recognised in the attentional test.

### Experiment #2: emotional semantic priming

The goal of Experiment #2 was to assess the effect of Emotional Semantic priming over DM. In this PIDM version, the task consisted of a total of 3 experimental and 2 control trials. During each trial, 4 faces were randomly presented 5 times, for 2000 ms (interspersed with a 1000 ms mask) and associated with a phrase with an emotional valence. According to their emotional association, four different categories of faces were assayed: Positive, Negative, Neutral or Mixed (2 positive, 2 negative, and 1 neutral phrases) (Fig. 3A).

The phrases (n = 152) were developed in collaboration with Federico Testoni (BA in Linguistics) in Spanish, with no more than seven (7) words each, and with political content (positive or negative) or not (neutral). To characterise them in terms of their emotional valence, 72 participants were asked to rate them on a 5-point Likert scale [from "Very unpleasant" to "Very pleasant"]. Three datasets of phrases (Positive, Negative or Neutral) were built, which the experiment code internally and randomly associated with each face category. Positive phrases were considered those whose mean was greater than 3.5, negative sentences were those with a mean less than 2.5, and neutral sentences were those with a mean between 3.5 and 2.5. Supplemental Table 2 shows the used phases (English translated version) and their scores. No sentence was repeated for each subject throughout the experiment.

Each experimental trial consisted of five presentations, each of 4 faces (randomly selected from the dataset of faces) associated with positive, negative or neutral phrases (randomly selected from each dataset) depending on the face category. Two different control trials were randomly intercepted between experimental trials. One control trial consisted of the presentation of 4 faces associated with only neutral phrases (Control #1) whereas the other (Control #2) of the presentation of 2 faces was associated with neutral phrases and 2 with the mixed condition (2 positive, 2 negative, and 1 neutral). This control was added to assess whether the balanced association with positive and negative emotional content is compensated and whether it induces an equivalent choice preference to neutral faces.

The experiments were developed in order to record: (1) the chosen face; (2) the valence category of the chosen face (Choice Preference); (3) its position in the randomly generated order; (4) the response time of the choice; (5) the confidence; and (6) the number of faces recognised in the attentional test.

### Experimental series #2: parametric conditions of repetition priming

In the second Experimental Series, two versions of Experiment #1 were run consecutively in random order. Experiment #1 was equivalent to the described above, but in the first version, each face presentation lasted 20 ms (Subliminal Condition), whereas in the second, they lasted 2000 ms (Lasting Condition) (Fig. 2A). In both versions, face presentations were interspersed with a 200 ms mask. Both versions of the experiments recorded: (1) the chosen face; (2) the valence category of the chosen face (Choice Preference); (3) its position in the randomly generated order; (4) the response time of the choice; (5) the confidence; and (6) the number of faces recognised in the attentional test.

### Experimental series #3: long-term priming effect

The purpose of this Experimental Series was to evaluate the persistence of priming in the long term. It consisted of two experimental sessions, separated by 24 h, performed in a synchronic condition (Fig. 4A). Although the experiments were carried out using the online platform, in this synchronic condition, participants were summoned for a video meeting in which the researcher informed them about the experiment. This experimental condition allowed us to control the environmental context and the emotional state in which participants found themselves (calmness, mood, etc.). The dynamics of the experimental session were maintained for the second day. During the experiments and the memory test, participants were offered to turn off their cameras and microphones.

In the first session (Day 1), participants performed experiments #1 and #2 as described for Experimental Series #1. The second session (Day 2) involved only the Testing Phase (Choice and Confidence) of each trial performed the day before. Thus, each participant was instructed to choose (depending on the experimental group) one face among the four presented the day before (for each trial) without passing through the Acquisition Phase again. The experiments were developed in order to record for each day: (1) the chosen face; (2) the frequency or the valence category of the chosen face (Choice Preference); (3) its position in the randomly generated order; (4) the response time of the choice; (5) the confidence; and (6) the number of faces recognised in the attentional test. Besides these variables, a Memory Ratio was calculated as the number of faces that were chosen again on day 2 over the total number of choices.

At the end of the second session, after the Testing phase, participants were asked to indicate the reasons for their choices on both days, from a list of options: Familiarity, Trust, Face Feature, Identification, Memory and other.

### Statistical analysis

All statistical analyses were performed in RStudio. The Choice Preference (frequency or emotional valence of the chosen face) was analysed using a Multinomial Logit Model, using the mlogit package^84^. Response Time was analysed using a Generalised Linear Model (GLM) or a Generalised Linear Mixing Model (GLMM), with a gamma distribution, using glmmTMB package^85^. Assumptions were checked using the DHARMa package^86^. Differences in confidence between the groups were explored by analysing the distribution of probabilities and the cumulative probabilities using the Kolmogorov–Smirnov Test^87^; significance of the differences in the cumulative probability distribution was assessed also by bootstrapping. The Memory Ratio was analysed using a Generalised Linear Model, with a Bernoulli distribution.

### Social study

The social study aimed to evaluate, from a more ecological task (an election of the Argentine president) and at a social level, the possible priming effect due to the press media on each candidate’s voting probability during the 2019 presidential election in Argentina. The Argentine Presidential Election consisted of a two-step election. The first step, named PASO [August 11th, 2019], is intended to filter the main presidential formulas to be presented in the General Election [Oct 27th, 2019]. Only candidates receiving more than 1.5% of the votes in the PASO elections advance to the General Election. The candidates who advanced to the General Election in 2019 were: M. Macri [MM], A. Fernandez [AF], R. Lavagna [RL], N. Del Caño [NDC], J. Gomez Centurion [JGC] and J. L. Espert [JLE] (Suppl. Table 6). MM was the official candidate (moderate right-wing), at the time of the election president of Argentina elected in 2015^30^. AF was the candidate of the majority opposition, an alliance of mainly peronist sectors (although it also includes non-peronist sectors), which governed Argentina during 2003–2015. NDC represents the minority left-wing opposition, while JGC and JLE represent the minority radical right-wing opposition. RL represents a moderate right-wing alternative.

Two online surveys were conducted in the periods prior to the PASO and the General Election (Suppl. Fig. 1) in order to study subjective variables (Familiarity, Trust and Voting Probability for each presidential formula/candidate) as well as the impact of the main national digital written media used to inform voters about candidates. In the period prior to the General Election, a news dataset of these main online written media was generated, to analyse the frequency of candidate mentions and their emotional associations (Suppl. Fig. 1).

### Surveys and subjective dataset

The online surveys were conducted in two periods of the electoral process: Period# 1 (pre-PASO; 23-06-2019 and 08-08-2019) and Period #2 (pre-General Election; 01-09-2019 and 24-10-2019). In both stages, the electoral ban was respected. The surveys were conducted using the Google Forms platform. Supplemental Table 3 summarises the different sections of the surveys, with the variables to analyse. Once personal and political characterisation of the participants was completed (Suppl. Table 3: Sections 2–3), the faces of the different presidential and vice presidential candidates and their political affiliation were randomly presented so that participants were able to recognize them or not, and to rank their familiarity, trust and voting probability on a 9-point Likert Scale (Section 4). Candidate images were extracted from campaign materials. Afterwards, they were asked for the Vote Reasons (Section 5) and the main means by which the survey population informed itself about the candidates (Section 6). To control automated answers, sham print/digital and audio-visual media were included; participants choosing any of these fake options were excluded from the analysis.

### Data acquisition and news dataset

In order to evaluate the frequency of mention of the different presidential formulas/candidates and their emotional associations, a bot was programmed with Python Language to collect the news published between 21 September and 27 October 2019 by the main national print media (Suppl. Table 4). The dataset generated included 22,510 newspaper articles, disaggregated by Publication Date, Press Media, Article Headlines and News Corpus. The complete News Dataset is available at https://osf.io/qbdrv/.

For the following analysis, different keyword combinations of candidates or presidential formulas were taken into account to avoid biased language used by different media, filter irrelevant information and increase the likelihood of capturing mentions. The keywords or phrases used to capture each candidate mention (as the candidate target) are summarised in Supplemental Table 5. Mentions of other candidates of the same political force (e.g., for a state governor) were not included in this analysis.

### Candidate mention frequency

The frequency of candidates’ mentions was calculated as the number of mentions of each candidate on the article corpus or the headlines. This information was extracted from the news dataset using tidy data principles in RStudio^88^. Both measures were calculated for each media source. For the purpose of our work, both measures are assumed to be sub-sampled parameters of voters' actual exposure to visual and semantic content related to each candidate. Once the measure of mention was obtained for each candidate for each media outlet, a weighted value EXP(p, c) was calculated for each pair of participant p and candidate c (according to the media outlets used for information), that represents the maximum possible exposure for the participant p to each candidate c (EXP_CORPUS and EXP_HEAD). For this purpose, the following formulation was used:

$$EXP\left( {p,c} \right) = \mathop \sum \limits_{{{\text{m}} \in {\text{M}}\left( {\text{p}} \right)}} {\text{E}}\left( {{\text{m}},{\text{c}}} \right)$$

where M(p) is the set of media outlets that the participant p declared to use to be informed about the candidates. E(m, c) is the maximum number of mentions in news articles mentioning the candidate c, published by the media outlet m.

### Sentiment analysis

Traditional Sentiment Analysis consists of polarity sentence-level analysis, assigning a polarity label (negative, neutral, or positive) to a whole sentence. For some instances of the collected data, however, mixed polarities could be extracted for different targets. This problem is usually called target-level sentiment analysis, in which a polarity label is assigned to a pair (text, target), where the target is present on the text. As no Spanish dataset was available for this task, we decided to build our own. For this purpose, we filtered the 2253 headlines mentioning at least one candidate from our news dataset. Three participants were recruited to rank the headlines according to whether they perceived them as favouring the candidates’ image (positive), disfavouring them (negative), or simply describing a fact (neutral). To reduce the ideological bias of the participants, the name of each presidential candidate/formula was replaced by a masked target (i.e., MM → Target C). A Labelling Manual was provided to the annotators, with instructions and examples of how to perform the task, and interviews were held with participants to consolidate the labelling criteria. Participants received $2000 as monetary compensation for the task. The agreement between the 3 participants was measured using the Krippendorff’s Alpha Coefficient, with a value greater than 0.4 considered acceptable. The number of positive, negative, or neutral labels was then calculated for each candidate and each media source. Once obtained, a weighted value POS(p, c) and NEG(p, c) were calculated for each pair of participant p and candidate c (according to the media outlets used for information), that represents the maximum possible exposure of positive or negative content for the participant p to each candidate c (POS_W and NEG_W). For this purpose, the following formulation was used:

$$POS\_W\left( {p,c} \right) = \frac{{\mathop \sum \nolimits_{{{\text{m}} \in {\text{M}}\left( {\text{p}} \right)}} {\text{POS}}\left( {{\text{m}},{\text{c}}} \right)}}{{\mathop \sum \nolimits_{{{\text{c}^{\prime}} \in {\text{C }}}} \mathop \sum \nolimits_{{{\text{m}} \in {\text{M}}\left( {\text{p}} \right)}} {\text{E}}\left( {{\text{m}},{\text{c}^{\prime}}} \right)}}$$

$$NEG\_W\left( {p,c} \right) = \frac{{\mathop \sum \nolimits_{{{\text{m}} \in {\text{M}}\left( {\text{p}} \right)}} {\text{NEG}}\left( {{\text{m}},{\text{c}}} \right)}}{{\mathop \sum \nolimits_{{{\text{c}^{\prime}} \in {\text{C }}}} \mathop \sum \nolimits_{{{\text{m}} \in {\text{M}}\left( {\text{p}} \right)}} {\text{E}}\left( {{\text{m}},{\text{c}^{\prime}}} \right)}}$$

where M(p) is the set of media outlets that the participant p declared to use to be informed about the candidates. C is the set of all candidates or political forces. POS(m, c) is the maximum number of positive targets in news headlines mentioning the candidate c, published by the media outlet m. NEG(m, c) is the maximum number of negative targets in news headlines mentioning the candidate c, published by the media outlet m. E(m, c) is the maximum number of mentions in news headlines mentioning the candidate c, published by the media outlet m.

### Statistical analysis

All statistical analyses were performed in RStudio, using the ordinal library. For analysis in a Multinomial Ordinal Model (ordinal package^89^), Voting Probability was considered as the response variable, and others as explanatory variables. Although different models were obtained, the final model was chosen on the basis of the variables that were significant and with the lowest Akaike Information Criterion (AIC). Both period datasets were separately analysed. In both cases, it was checked that the assumptions were met. Relation between variables were analysed by Spearman correlations, and the means of each variable for each candidate (for Period #2) were also analysed by a Spearman cross-correlation with themselves, with the campaign expenses (published in the National Electoral Directorate: https://www.argentina.gob.ar/interior/dine) and the election results as the percentage of vote.

### Ethical approval

The study and all methods were carried out in accordance with the relevant guidelines and regulations, previously approved by the ethic committee of the Clinic Hospital “José de San Martín”, of the University of Buenos Aires. The study complies with the principles of the Declaration of Helsinki. All participants gave informed consent to participate in experiments or surveys, also informed consent was obtained from all subjects for publication of identifying images or information in an online open-access publication. Identifying information was removed from datasets and replaced by an anonymous ID.

## Supplementary Information

Supplementary Information.

## Data Availability

The scripts and datasets generated and/or analysed during the current study are available in the OSF repository, https://osf.io/qbdrv/.
