# Supplementary material for: Top-down modulation impairs priming susceptibility in complex decision-making with social implications
Source: Sci Rep. 2022 Oct 25;12:17867. doi: 10.1038/s41598-022-22707-x (PMC9595095; doi:10.1038/s41598-022-22707-x)
Supplement: Supplementary file 1 — Supplementary Information. [file 41598_2022_22707_MOESM1_ESM.pdf]

Supplemental Material

Title:
Top-Down Modulation Impairs Priming Susceptibility in Complex Decision-Making with Social Implications

Extended Results

Choice Preference and Response Time models

The Choice Preference (CP) was analysed as the Frequency or Emotional valence of the chosen face, using a Multinomial Logit Models (MLM). Models were built including variables with low VIF (low multicollinearity). In all experiments, no evidence was observed that the Independence of Irrelevant Alternatives (IIA) assumption is not met, tested with a Hausman-McFadden test. There was also no evidence that the model does not fit a logistic regression, tested by Hosmer-Lemeshow tests.

The Response Time (RT) was analysed using a Generalised Linear Model (GLM) or Generalised Linear Mixing Models (GLMM), with a gamma distribution. In all experiments (unless otherwise clarified), no evidence was observed that assumptions are not met or that the model does not fit a logistic regression, tested with KS, Dispersion and Outlier tests, using DHARMa package.

In Experimental Series #3, Memory Ratio [MR, estimated as the number of faces that were chosen again on day 2 over the total number of choices] was analysed using a Generalised Linear Model (GLM), with a Bernoulli distribution.

Experiment #1 (Experimental Series #1): A total of 93 adults participated in Experiment #1, of which 71 were included in the analysis (ages 20-73, mean 34.85 ± 14.2; 34 women; Supp. Table 1).

The CP model was built including GROUP, AGE, GENDER and EDUCATION as explanatory variables. Only GROUP showed a significant effect (See Results). No significant effect was observed for AGE [LR Chisq = 4.79, p = 0.18], GENDER [LR Chisq = 6.12, p = 0.10] and EDUCATION [LR Chisq = 20.17, p = 0.32].

The RT model included FREQ, GROUP (and interaction FREQ:GROUP), AGE, GENDER and EDUCATION as explanatory variables. Only GROUP (see Results) and FREQ [LR Chisq = 12.16, p = 0.0068] showed a significant effect. However, no significant effect was observed for the interaction FREQ:GROUP [LR Chisq = 3.04, p = 0.38], nor AGE [LR Chisq = 3,45, p = 0.06], GENDER [LR Chisq = 0.97, p = 0.32] or EDUCATION [LR Chisq = 7,98, p = 0.23]. In the case of the Control trial, the RT model included GROUP, AGE, GENDER and EDUCATION as explanatory variables: again, only GROUP showed a significant effect (see Results; Fig. 1E); AGE [LR Chisq = 0.13, p = 0.71], GENDER [LR Chisq = 0.16, p = 0.68] and EDUCATION [LR Chisq = 6.38, p = 0.38] did not show significant effects.

Experiment #1 (Experimental Series #2): On the other hand, a total of 101 adults participated in this Experimental Series, of which 69 were included in the analysis of subliminal condition (ages 18-75, mean 35.7 ± 14.4; 42 women) and 76 in the lasting condition (ages 18-75, mean 37.9 ± 15.6; 48 women).

Both CP models were built including only GROUP and AGE as variables (GENDER and EDUCATION had high multicollinearity). For the subliminal condition (SC), only GROUP showed a significant effect (See Results), but AGE did not [LR Chisq = 1.22, p = 0.74]. For the lasting condition (LC), neither GROUP (see Results) nor AGE [LR Chisq = 1.63, p = 0.65] showed significant effects.

Both RT models included FREQ, GROUP (and interaction FREQ:GROUP), AGE, GENDER and EDUCATION as explanatory variables. GROUP showed a significant effect in both conditions (see Results). Besides, AGE was also significant in the SC [LR Chisq = 10,06, p = 0.0015], but not in the LC [LR Chisq = 3.08, p = 0.078] No significant effect was observed for the interaction FREQ:GROUP [SC: LR Chisq = 0.81, p = 0.84; LC: LR Chisq = 1.41, p = 0.70], GENDER [SC:

LR Chisq = 1.82, p = 0.40; LC: LR Chisq = 0.19, p = 0.90] or EDUCATION [SC: LR Chisq = 4.96, p = 0.66; LC: LR Chisq = 4.98, p = 0.66]. In the case of the Control trials, the RT models included GROUP, AGE, GENDER and EDUCATION as explanatory variables: again, only GROUP showed a significant effect (see Results; Fig.2D,H); AGE [SC: LR Chisq = 2.92, p = 0.08; LC: LR Chisq = 2.69, p = 0.10], GENDER [SC: LR Chisq = 0.33, p = 0.84; LC: LR Chisq = 3.27, p = 0.19] and EDUCATION [SC: LR Chisq = 13.38, p = 0.06; LC: LR Chisq = 6.30, p = 0.50] did not show significant effects.

Experiment #2 (Experimental Series #1): A total of 93 adults participated in Experiment #2, of which 66 were included in the analysis (ages 20-77, mean 35.10 ± 14.58; 36 women; Supp.Table 1).
The CP model was built including GROUP, AGE, and GENDER as explanatory variables. Only AGE showed a significant effect [LR Chisq = 14.37, p = 0.002]. No significant effect was observed for GENDER [LR Chisq = 6.83, p = 0.07] and GROUP (see Results). In the case of Control #2, no significant effect was observed in the model built including GROUP [LR Chisq = 0.0009, p = 0.97] (Fig.3E), AGE [LR Chisq = 0.53, p = 0.46], GENDER [LR Chisq = 1.75, p = 0.18] and EDUCATION [LR Chisq = 9.30, p = 0.15]. We speculated on 3 possible scenarios. In the first, if the priming power were equivalent but reversed, there would not be any difference between the preference of the mixed and neutral faces. If it was not, a greater or lower preference for the mixed would suggest that the positive or negative valence of the phrases has a greater priming power, respectively. Our results suggest the first possibility (Fig.3E).

The RT model included VAL, GROUP (and interaction VAL:GROUP), AGE, GENDER and EDUCATION as explanatory variables. Only VAL showed a significant effect (see Results). No significant effect was observed for GROUP (see Results), the interaction VAL:GROUP [LR Chisq = 0.78, p = 0.85], AGE [LR Chisq = 0.94, p = 0.33], GENDER [LR Chisq = 3.45, p = 0.06] or EDUCATION [LR Chisq = 7,20, p = 0.30]. In the case of the Control #1, none of the included variables to build the RT model was significant: GROUP [LR Chisq = 0.64, p = 0.42] (Fig.3D), AGE [LR Chisq = 0.12, p = 0.72], GENDER [LR Chisq = 0.75, p = 0.38] and EDUCATION [LR Chisq = 3.43, p = 0.75].
In the case of the Control #2, the RT model included GROUP, VAL, interaction VAL:GROUP, AGE, GENDER and EDUCATION as explanatory variables. Only VAL showed a significant effect [LR Chisq = 5.57, p = 0.018] (Fig.3F; GROUP [LR Chisq = 0.37, p = 0.53], VAL:GROUP [LR Chisq = 3.50, p = 0.06], AGE [LR Chisq = 0.33, p = 0.24], GENDER [LR Chisq = 1.84, p = 0.17] and EDUCATION [LR Chisq = 3.55, p = 0.73] did not show significant effects.

Experimental Series #3: In order to evaluate the persistence of the priming effect, we conducted Experimental Series #3. This series consisted of a two-session synchronic experiment, online but monitored by the researcher via video meeting. A total of 48 adults participated in this experimental series, of which 39 were included in the analysis for Experiment #1 (ages 18-44, mean 26.6 ± 6.9; 32 women; Supp.Table 1) and 43 were included in the analysis for Experiment #2 (ages 18-48, mean 27.5 ± 7.4; 33 women; Supp.Table 1).

Experiment #1: For day 1, the CP model was built including GROUP and AGE as explanatory variables. No significant effect was observed for GROUP (see Results) and AGE [LR Chisq = 2.59, p = 0.45]. For day 2, the CP model only included GROUP; no significant effect was observed [LR Chisq = 2.09, p = 0.55]. CP models were built by separate groups to analyse differences between days (including only DAY as explanatory variable): significant differences were observed for UST [LR Chisq = 13.188, p = 0.004] and IMT [LR Chisq = 16.623, p = 0.0008] groups.
The MR model included GROUP, FREQ 1 (the frequency of the face chosen the 1st day), FREQ 2 (the frequency of the face chosen the 2nd day), AGE, GENDER and EDUCATION. No significant effect was observed: GROUP [LR Chisq = 0.02, p = 0.87], FREQ1 [LR Chisq = 4.79, p = 0.18], FREQ2 [LR Chisq = 5.44, p = 0.14], AGE [LR Chisq = 0.20, p = 0.64], GENDER [LR Chisq = 2.92, p = 0.23] and EDUCATION [LR Chisq = 4.56, p = 0.60].

The RT model included FREQ, GROUP (and interaction FREQ:GROUP), AGE, GENDER and EDUCATION as explanatory variables. Only GROUP (see Results) and the interaction FREQ:GROUP [Chisq = 10.28, p = 0.0013] showed a significant effect. However, no significant effect was observed for FREQ itself [Chisq = 0.86, p = 0.83], nor AGE [Chisq = 0.73, p = 0.39], GENDER [Chisq = 0.10, p = 0.94] or EDUCATION [Chisq = 4.52, p = 0.60]. In the case of day 2, none of the included variables showed a significant effect: FREQ [LR Chisq = 0.70, p = 0.87], GROUP [LR Chisq = 2.86, p = 0.09] or FREQ:GROUP [LR Chisq = 1.41, p = 0.70]. RT models were built by separate groups to analyse differences between days (including only DAY, FREQ and their interaction as explanatory variables): no differences were observed for IMT [DAY: Chisq = 2.53, p = 0.11; FREQ: Chisq = 2.72, p = 0.43; FREQ:DAY: Chisq = 1.33, p = 0.72], thought UST was significant for DAY [DAY: Chisq = 6.93, p = 0.008; FREQ: Chisq = 1.76, p = 0.62; FREQ:DAY: Chisq = 1.58, p = 0.66]. Respect to the Control trials, the model included GROUP, AGE, GENDER and EDUCATION as explanatory variables. For day 1, only GROUP showed a significant effect [LR Chisq = 8.37, p = 0.003] (Fig. 4E); AGE [LR Chisq = 1.73, p = 0.18], GENDER [LR Chisq = 2.57, p = 0.27] and EDUCATION [LR Chisq = 5.22, p = 0.351] did not show significant effects. For day 2, GROUP [LR Chisq = 10.78, p = 0.001] and AGE [LR Chisq = 5.44, p = 0.019] showed a significant effect (Fig. 4E), but not GENDER [LR Chisq = 2.28, p = 0.31] and EDUCATION [LR Chisq = 8.66, p = 0.19] did not show significant effects.

Experiment #2: For day 1, the CP model was built including GROUP and AGE as explanatory variables. No significant effect was observed for GROUP [LR Chisq = 4.95, p = 0.17] and AGE [LR Chisq = 1.44, p = 0.69]. For day 2, the CP model only included GROUP, which also showed no significant effect [LR Chisq = 1.10, p = 0.77]. CP models were built by separate groups to analyse differences between days (including only DAY as explanatory variable): a significant "DAY" effect was observed for both UST [LR Chisq = 13.188, p = 0.004] and IMT [LR Chisq = 16.623, p = 0.0008] groups.
The MR model included GROUP, VAL1 (the valence of the face chosen the 1st day), VAL2 (the valence of the face chosen the 2nd day), AGE, GENDER and EDUCATION. A significant effect was observed for VAL2 (see Results) and GENDER [LR Chisq = 8.60, p = 0.01], but not for GROUP [LR Chisq = 0.23, p = 0.62], VAL1 [LR Chisq = 3.33, p = 0.34], AGE [LR Chisq = 0.00, p = 0.99], and EDUCATION [LR Chisq = 2.69, p = 0.84].
The RT model included VAL, GROUP, AGE, GENDER and EDUCATION as explanatory variables. Only GROUP (see Results) and VAL [Chisq = 20.64, p = 0.0001] showed a significant effect. However, no significant effect was observed for AGE [Chisq = 0.61, p = 0.43], GENDER [Chisq = 1.01, p = 0.60] or EDUCATION [Chisq = 2.78, p = 0.83]. In the case of day 2, none of the included variables showed a significant effect: VAL [LR Chisq = 7.03, p = 0.07], GROUP [LR Chisq = 0.37, p = 0.53] or VAL:GROUP [LR Chisq = 1.51, p = 0.67]. RT models were built by separate groups to analyse differences between days (including only DAY and VAL as explanatory variables). As DAY as VAL showed significant effect for both IMT [DAY: Chisq = 18.03, p = 2.16e-05; VAL: Chisq = 9.36, p = 0.02], and UST [DAY: Chisq = 40.53, p = 1.93e-10; VAL: Chisq = 11.69, p = 0.008]. Respect to the Control trials, the model included GROUP, AGE, GENDER and EDUCATION as explanatory variables. For day 1, no significant effect was observed: GROUP [LR Chisq = 1.19, p = 0.27] (Fig. 4J); AGE [LR Chisq = 0.02, p = 0.88], GENDER [LR Chisq = 2.34, p = 0.30] and EDUCATION [LR Chisq = 5.21, p = 0.51]. For day 2, GROUP showed a significant effect [LR Chisq = 4.61, p = 0.03] (Fig. 4J), but not AGE [LR Chisq = 0.14, p = 0.70], GENDER [LR Chisq = 0.13, p = 0.93] and EDUCATION [LR Chisq = 5.36, p = 0.49].

Primacy and Recency

For Experimental Series #1, we analysed the data to assess whether subjects chose the faces presented in the first or last positions (Primacy or Recency effect, respectively). The Supplementary Figure 2A-B shows histogram plots as a function of the first or last appearance position of the chosen face in the experimental trials (disaggregated by chosen frequency or valence) and control trials. The data were analysed using a Generalised Linear Model, with a quasipoisson distribution. For Experiment #1, an analysis of Deviance showed no primacy or recency effect by GROUP [PRIMACY: LR Chisq = 0.45, p = 0.50; RECENCY: LR Chisq = 0.05,

p = 0.82] but it did by FREQ [PRIMACY: LR Chisq = 445.32, p = 2e-16; RECENCY: LR Chisq = 416.97, p = 2e-16]. The dispersion parameters (DP) for quasipoisson family were 1.41 and 0.97 for PRIMACY and RECENCY analysis, respectively. This result can be explained by the fact that the chosen faces associated with first or last positions correspond mainly to faces that were shown 12 times. If the choice of the UST group could have been explained by a primacy or recency effect, then there should be a significant group effect. The analysis of the control confirms this result [PRIMACY: LR Chisq = 0.108, p = 0.74, DP = 1.38; RECENCY: LR Chisq = 0.05, p = 0.81, DP = 0.39].
For Experiment #2, no primacy or recency effects were observed by analysing the variables GROUP [PRIMACY: LR Chisq = 0.0019, p = 0.96; RECENCY: LR Chisq = 0.48, p = 0.48] or VAL [PRIMACY: LR Chisq = 1.07, p = 0.78; RECENCY: LR Chisq = 0.41, p = 0.93]. DPs were 1.84 and 0.34 for PRIMACY and RECENCY analysis, respectively. The analysis of the control #1 confirms this result [PRIMACY: LR Chisq = 0.50, p = 0.47, DP = 1.72; RECENCY: LR Chisq = 0.02, p = 0.86, DP = 0.38]. The analysis of the control #2 allowed to check these effects not only regarding to GROUP but also VAL (comparing NEU versus MIX condition): no significant primacy [GROUP: LR Chisq = 3.16, p = 0.07; VAL: LR Chisq = 0.28, p = 0.59; DP = 1.70] or recency [GROUP: LR Chisq = 0.02, p = 0.87; VAL: LR Chisq = 0.003, p = 0.95; DP = 0.40] effects were observed.

Confidence analysis

Confidence was assessed using a 9-point Likert scale. An exploratory analysis showed no significant differences in mean confidence between frequency (Experiment #1) and valence (Experiment #2) or groups. However, distributional differences are usually not detectable by analysis of the mean or median; therefore, in this case we preferred to statistically assess differences in the distributions of confidence, using the cumulative probability and the Kolmogorov–Smirnov (KS) test. Significant differences were also analysed by bootstrapping.

In Experiment #1 (Experimental Series #1), different distributions of reported confidence values were observed for each group, showing a trend towards lower confidence in the IMT group. The KS test revealed a greater distance of Kolmogorov–Smirnov (D = 0.23153) between both cumulative distributions, being significant for the test (p = 0.006) and by bootstrapping (p = 8.00e-4) (Fig.1F). For Experimental Series #2, significant differences between cumulative distributions of both groups were observed only under the Lasting Condition (2000ms): the distance of KS (D = 0.1684) was only significant by bootstrapping (p = 0.02) but not significant by the KS test (p = 0.07) (Fig.2I). For Experiment #1 of Experimental Series #3, no significant differences between cumulative distributions of both groups were observed (Fig.4F), probably because these experiments had a smaller number of subjects due to the fact that they were synchronic and involved two days.

In Experiment #2, no significant differences between cumulative distributions of both groups were observed (Fig.3G). For the synchronic condition, no significant differences were detected on day 1, but they were on day 2 [KD = 0.29, p = 0.01; bootstrapping: p = 0.002) (Fig.4K).

Social Study

As described in the Results section, the Argentine Presidential Election consisted in a two-step's elections. The first step, PASO, was dated on August 11th, 2019, and 10 candidates and their political force or coalition were presented (Suppl. Table 6). The aim of this first election is to filter the main presidential formulas to be presented in the General Election by reaching more than 1.5% of the votes. Thus, at the General Election (October 27th, 2019), only 6 candidates were presented: Mauricio Macri [MM], Alberto Fernandez [AF], Roberto Lavagna [RL], Nicolas Del Caño [NDC], Jorge Gomez Centurion [JGC] and Jose Luis Espert [JLE] (Suppl. Table 6).
For the purpose of the Social Study, we conducted online surveys during June 22th to August 11th [Period #1] and August 12th to October 27th [Period #2], to assess the Familiarity, Trust and Voting Probability for each candidate, as well as the social and press media the participants used to get candidate information (Supp. Table 3). For Period #1, the survey was completed by 2255 participants, of which only 2188 were included in the analysis (of which 44 were between 16 and 17 years old, the age at which voting is optional in Argentina; 1202 women). For Period #2, the

survey was completed by 1418 participants, of which only 1398 were included in the analysis (of which 16 were between 16 and 17 years old, the age at which voting is optional in Argentina; 835 women).

Variables were analysed by Multinomial Ordinal Model. For both periods, Voting Probability was considered the response variables, while others explanatory variables. In a first instance, rows containing NA data were removed from the original dataset (it is important to clarify that this did not necessarily imply the removal of entire subjects). From the 19700 rows of the dataset of Period #1, a total of 1283 rows were deleted (6.51%); in case of Period #2, from the 8388 rows of the dataset, a total of 674 rows were deleted (8.03%). For exploratory analysis, firstly a complete model (with all variables and no interaction) was performed in order to define which variables were significant to explain Voting Probability. Supplemental Figure 3A shows the Odds Ratios of all variables (their significance indicated by the respective asterisks) from Period #1; while Fig. 5A shows the Odds Ratios for the final model, including the significant variables. The final model was chosen as the one with lowest Akaike Information Criterion (AIC). No evidence was observed that the proportional odds assumption is not met. As described in the Results section, for Period #1, Trust and Familiarity mostly explain the variability of Voting Probability for each candidate [Odds Ratios (CI: 2.5%-9.75%): Trust: 1.95 (1.75-2.16); Familiarity: 1.31 (1.18-1.46)]. The impact of each candidate *per se*, or the Trust or Familiarity for each candidate per se, depend on each candidate, suggesting that main candidates (AF and MM) could have a higher effect on the final result. Another relevant variable was the Political Self-Perception, where the levels "apolitic" and "politic" had opposite effects (Fig.5A). By a Spearman correlation analysis, Voting Probability showed to correlate significantly with Trust [Spearman coeff. (ρ) = 0.82] and Familiarity [ρ = 0.56] (Fig.5B).

During the Period #2, besides the online survey, 22,500 newspaper articles, published between September 21 and October 27 in the main written media, were collected to generate a News Dataset. Frequency mention to each candidate in the corpus or headline of the news articles published by each main written media was assessed using Text Mining tools in R. We assumed that these measures are sub-samples of the total exposure to each candidate information. In order to evaluate the positive or negative association of each candidate mention in the headlines, sentiment analysis of headlines mentioning at least one candidate was performed. For this purpose, three participants targeted the headlines as positive, negative or neutral (for each candidate), with respect to whether they perceived it as favouring the candidate's image (positive), disfavouring (negative) or simply describing a fact (neutral) (Online Methods). Participant´s agreement was evaluated by Krippendorff's alpha: all candidates showed acceptable K´alphas (>0.4), except for JGC [what could be explained by the low number of headlines that mentioned him]. The analysis of the News Dataset revealed an asymmetry in the mentions of each candidate (Fig.5C) as well as in the positive or negative perception of their headlines (Fig.5D). Our analysis allowed us to calculate the frequency of mentions and the positive, negative or neutral perception of mentions in the headlines for each candidate and for each media outlet. Taking into account that in survey each participant reported consulting different media to obtain information about the candidates, we were able to calculate the maximum frequency of exposure of each candidate (and their positive or negative perception) for each participant including these variables (EXP_CORPUS, EXP_HEAD, POS_W, NEG_W) in the original dataset. Supplemental Figure 3B shown the Odds Ratios of all variables (their significance indicated by the respective asterisks) from Period #2 (complete model); while Figure 5E shows the Odds Ratios for the final model, including only the significant variables. An interesting point to note is that the variables EXP_CORPUS and EXP_HEAD were not significant and therefore were not included in the final model. In addition, Familiarity did not show a large effect on the Voting Probability [OR = 1.04] unlike in the first period. When variable relation was analysed by Spearman correlation, Familiarity significantly correlated with EXP_CORPUS [ρ=0.37] and EXP_HEAD [ρ = 0.38]. On contrast, weighted positive and negative perception showed to have great impact on Voting Probability [OR(POS_W) = 3.02 (2.03-4. 47); OR(NEG_W) = 0.28 (0.13-0.59)] (Fig.5F) and significantly correlated with Trust [ρ(POS_W) =0.28; ρ(NEG_W) = -0.11]. In turn, Trust showed to have a greater Odds Ratio [OR = 2.3] in comparison with Period #1.

Finally, cross-correlation analysis was performed to evaluate variable means per candidate, campaign expenses and electoral results (Suppl. Table 6). This analysis allows to evidence that candidates with lower Familiarity or Trust showed also lower Voting Probability. Besides, it supports the previous results about the relationship between the Familiarity and the exposure to candidate information, even accompanied by campaign expenses [$\rho = 0.90$; $p=0.012$] (Fig. 5G). Besides, Trust also correlated significantly with positive mentions (in this case, expressed as the total positive mentions of each candidate: POS_T).

Author contributions

FAB, TAS and PNFL designed the PIDM paradigm and Social Study Surveys. AB developed the digital platform [www.experimentoscognitivos.com] where experiments were conducted and the experiments that ran inside the platform. FAB conducted most cognitive experiments, and AC some control experiments. TAS, FAB and MG conducted the statistical analysis. DF programmed the bot that collected the news to generate the News Dataset; PNFL filtered and unified the criteria in the dataset. ER and JMP performed the sentiment analysis of news headlines. PNFL wrote the manuscript. All authors participated in reaching the final version of the manuscript.

Supplemental Figures

Supplemental Figure 1.

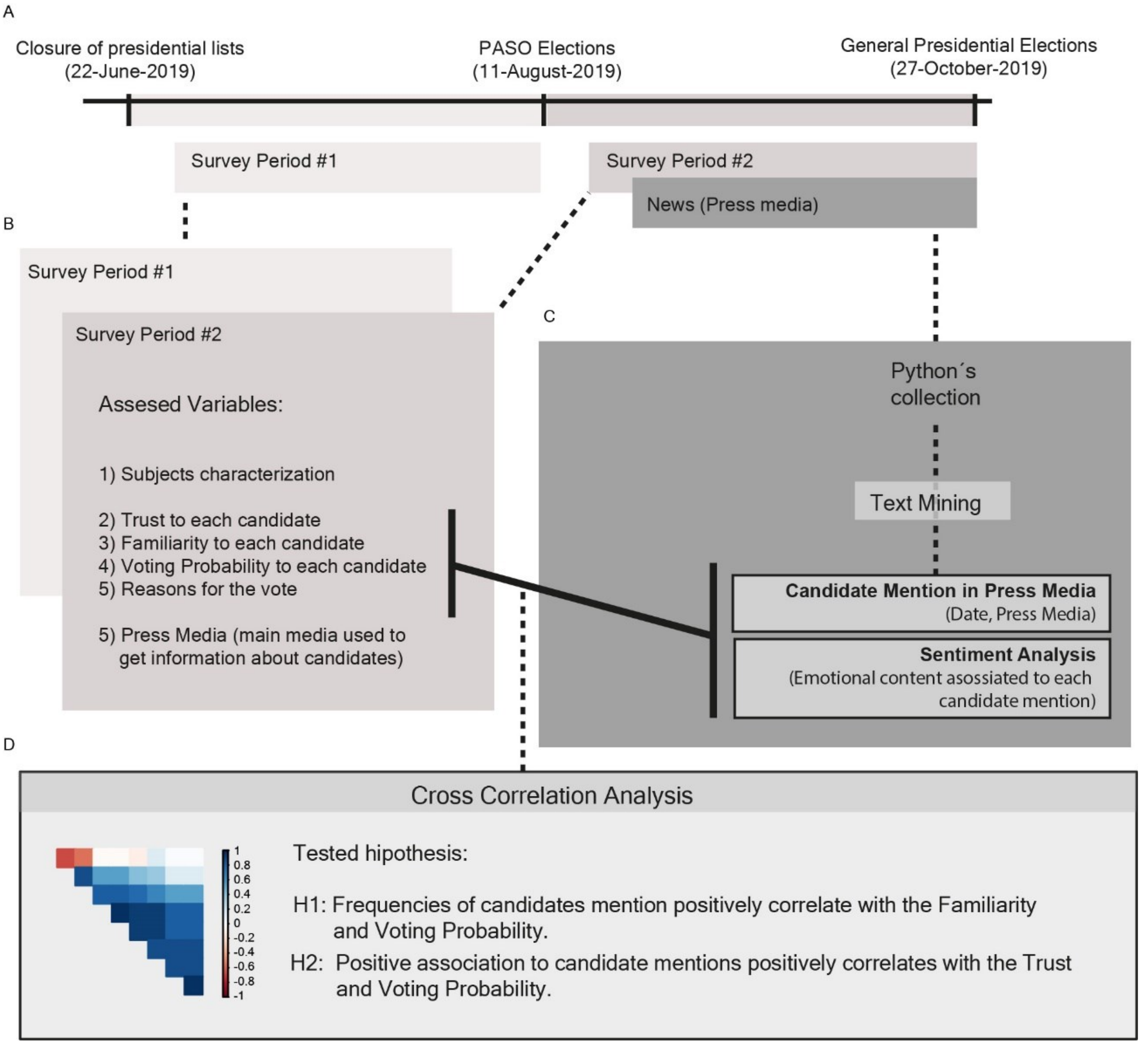

Supplemental Figure 1. Social Study design. A. Timeline of the election, including the period of surveys and news scraping in the main media outlets. B. Assessed variables of the surveys for the generation of the subjective dataset. C. News collection for the generation of the news dataset: analysis of mention frequency by text mining algorithms and sentiment analysis. D. Cross-correlation analysis between both datasets to contrast the working hypotheses.

Supplemental Figure 2.

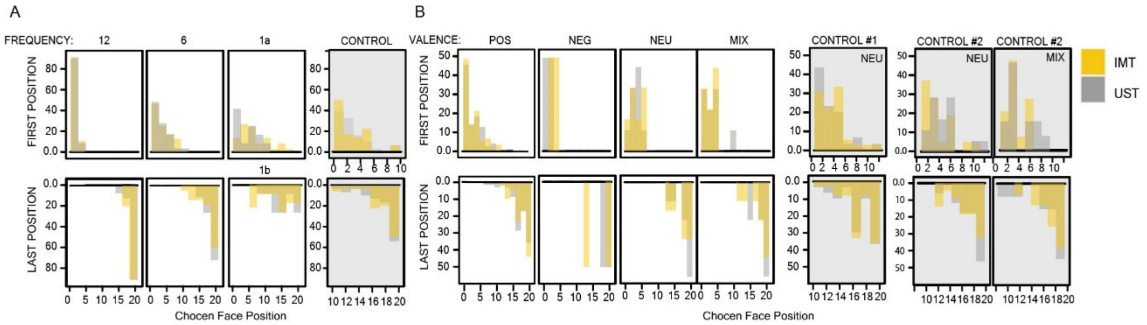

Supplemental Figure 2. *Histogram distribution of the chosen face, regarding the first position of appearance (above) or last position (below), desegregated by group, and analysed by frequency or valence, and Control trials, during Experiment #1 (A) and during Experiment #2 (B). *p<0.05, **p<0.01*

Supplemental Figure 3.

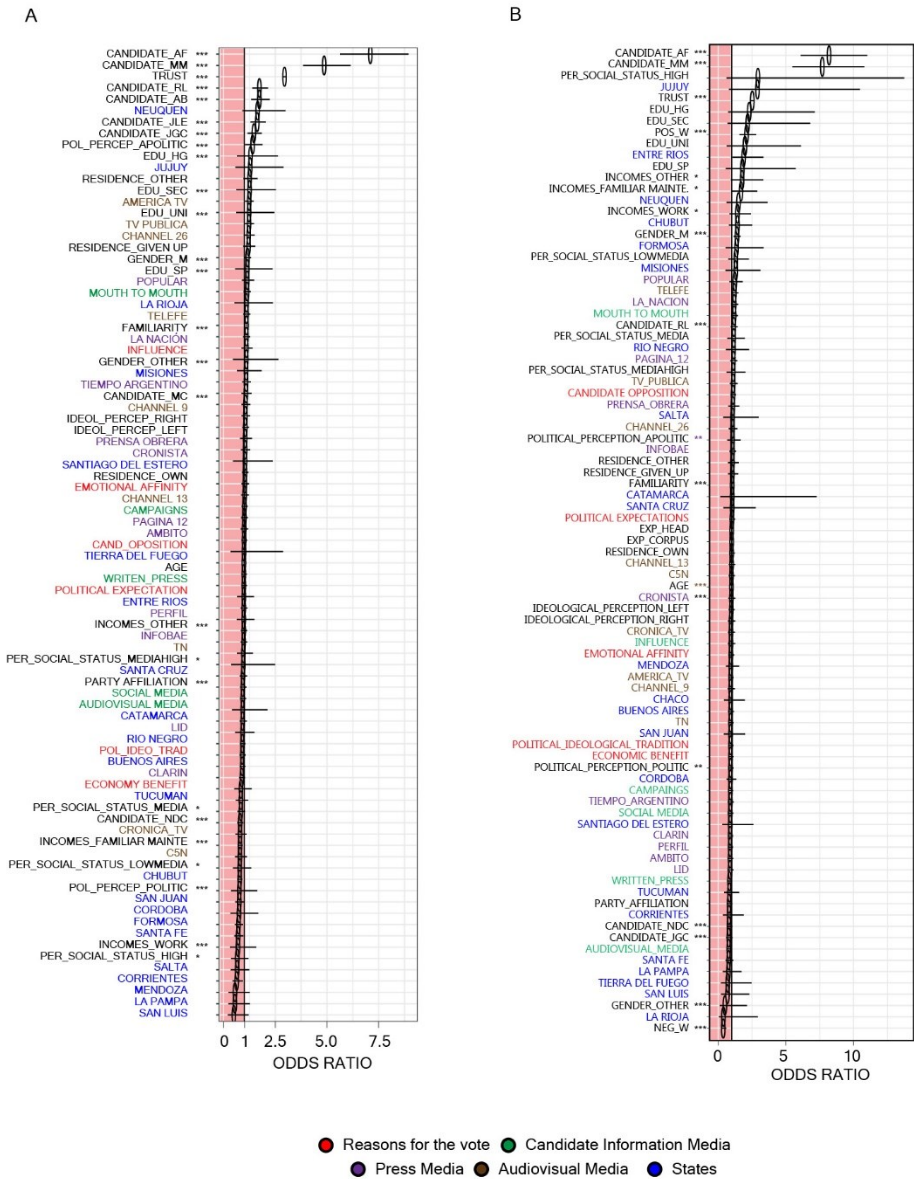

Supplemental Figure 3. *Complementary results of the Social Study. Odds ratios ± confidence intervals of the complete model with all variables for Period#1 (A) or Period#2 (B); significant variables are signed with respective asterisk. Variables are grouped by colour signalling type of variable: in red, reason for the vote [PIT: political ideological tradition; CO: candidate opposition; EB: economic benefit; EA: emotional affinity; I: influence]; in green, medias by which participants inform about candidate; in violet, the written media used; in brown, the audio-visual media; in blue, the Argentina provinces where participants live; in black, the other variables. AF: Alberto Fernandez; MM: Mauricio Macri; RL: Roberto Lavagna; NDC: Nicolas Del Caño; JGC: Jorge Gomez Centurion; JLE: Jose Luis Espert; MC: Manuela Castañeira; AB: Alejandro Biondini. POS_W and NEG_W: weighted positive and negative perception for each participant according to the media they use to get candidate information. POL_PERCEP: political self-perception, as POLITIC or APOLITIC person. GENDER_M (male). EXP_corpus or EXP_head: weighted mention in news corpus or headlines for each participant according to the media they use to get candidate information* $p<0.05$, ** $p<0.01$, *** $p<0.001$.*

Supplemental Tables

Supplemental Table 1. Population description

COGNITIVE EXPERIMENTS				
Population	Participants	Age (min-max)	n women	Reasons for exclusion
		mean±SEM	n men	
			n other	
1. Experimental Series #1				
Total	93	20 -77	41 (w)	
		34,7 ± 14,5	52 (m)	
1.a. Experiment #1				
Analysed	71	20-73	34 (w)	
		34,8±14,2	37 (m)	
Excluded	22	23 -77	7 (w)	Medication & Drugs - Response Time
		34,4 ± 15,5	15 (m)	Inattention criteria
1.b. Experiment #2				
Analysed	66	20 - 77	36 (w)	
		35,1 ± 14,7	30 (m)	
Excluded	27	23 - 69	5 (w)	Medication & Drugs - Response Time
		33,8 ± 14,1	22 (m)	Inattention criteria
2. Experimental Series #2				
Total	101	18 - 75	1 (o)	
		39,2 ± 16,2	60 (w)	
			40 (m)	
2.a. Experiment #1: 20ms				
Analysed [20ms	69	18 - 75	1 (o)	
		35,7 ± 14,4	42 (w)	
			26 (m)	
Excluded [20ms	32	19 - 72	18 (w)	Medication & Drugs - Response Time
		46,6 ± 17,5	14 (m)	Inattention criteria
2.b. Experiment #1: 2000ms				
Analysed [2000	76	18 - 75	1 (o)	
		37,9 ± 15,6	48 (w)	
			27 (m)	
Excluded [2000	25	19 - 72	12 (w)	Medication & Drugs - Response Time
		42,7 ± 18,1	13 (m)	Inattention criteria
3. Experimental Series #3				
Total	48	18 - 48	1 (o)	
		27,5 ± 7,5	36 (w)	
			11 (m)	
3.a. Experiment #1				
Analysed	39	18 - 44	1 (o)	
		26,6 ± 6,9	32 (w)	
			6 (m)	
Excluded	9	21 - 48	4 (w)	Inattention criteria
		31,33 ± 9,03	5 (m)	Response time
3.b. Experiment #2				
Analysed	43	18 - 48	1 (o)	
		27,5 ± 7,4	33 (w)	
			9 (m)	
Excluded	5	21 - 33	3 (w)	Inattention criteria - Medication & Drugs
		26,2 ± 4,7	2 (m)	Response time

SOCIAL STUDY				
Population	Participants	Age (min-max)	n women	Reasons for exclusion
		mean±SEM	n men	
			n other/na	
Period #1				
Total	2255	13-93	1002 (w)	
		34.2 ± 0.3	1234 (m)	
			19 (o / na)	
Analysed	2188 [1]	16-93	969 (w)	
		34.6 ± 0.3	1202 (m)	
			17 (o / na)	
Excluded	67	13-70	33 (w)	Scramble*, age under 16 years old
		22.7 ± 1.5	32 (m)	
			2 (o / na)	
Period #2				
Total	1418	11-88	843 (w)	
		37.6 ± 0.5	566 (m)	
			9 (o / na)	
Analysed	1398 [2]	16-88	835 (w)	
		37.7 ± 0.4	555 (m)	
			8 (o / na)	
Excluded	20	11-62	8 (w)	Scramble* , age under 16 years old
		30.8 ± 4.0	11 (m)	
			1 (o / na)	

[1] ≃2% (44 individuals) between 16 and 17 years old (age group not obliged to vote).
[2] ≃1% (16 individuals) between 16 and 17 years old (age group not obliged to vote).
*Individuals who chose a false information media.

Supplemental Table 2. Phrases used in Experiment #2 and their rated emotional valence

PHRASES	MEAN	SEM
Involved in human trafficking.	**1.03**	**0.02**
Justified trigger-happy behaviour.	**1.35**	**0.09**
Decreased the education budget.	**1.35**	**0.08**
Decreased the public health budget.	**1.36**	**0.08**
Closed community kitchens in deprived neighbourhoods.	**1.36**	**0.06**
Privatised national territories.	**1.36**	**0.08**
Increased repression in their government.	**1.38**	**0.08**
Decreased the science budget.	**1.41**	**0.08**
Increased unemployment during their time in power.	**1.43**	**0.08**
Censored opposition media.	**1.45**	**0.1**
Decreased the minimum wage.	**1.46**	**0.1**
Increased poverty during their time in power.	**1.46**	**0.09**
Decreased retirement pensions.	**1.48**	**0.07**
Encouraged the use of toxic agro-chemicals.	**1.48**	**0.08**
Is against the legalisation of abortion.	**1.5**	**0.1**
Participated in illegal acts.	**1.5**	**0.09**
Was prosecuted for acts of corruption.	**1.58**	**0.13**
Discouraged the development of national industry.	**1.59**	**0.11**
Worked half the working days.	**1.59**	**0.09**
Nationalised private debt.	**1.61**	**0.11**

Increased working hours.	**1.72**	**0.1**
Has offshore accounts.	**1.74**	**0.1**
Denied salary increases in bargaining.	**1.75**	**0.09**
Is against the secular state.	**1.78**	**0.13**
Raised taxes.	**1.85**	**0.08**
Eased restrictions on mining companies.	**1.85**	**0.13**
Raised the retirement age.	**1.95**	**0.12**
Took on debts to the imf.	**2.05**	**0.13**
Is against marijuana legalisation.	**2.09**	**0.11**
Had a negative trade balance while in power.	**2.09**	**0.09**
Removed subsidies on basic goods.	**2.09**	**0.13**
Aggressively debated with the opposition.	**2.22**	**0.11**
Never travelled abroad.	**2.59**	**0.08**
Clamped down on the dollar.	**2.63**	**0.13**
Decreased state jobs.	**2.68**	**0.14**
Can't swim.	**2.74**	**0.07**
Suffered a fracture when they were young.	**2.75**	**0.07**
Had knee surgery.	**2.8**	**0.07**
Their favourite colour is brown.	**2.86**	**0.07**
Have an allergy to cats.	**2.87**	**0.07**
Lowered tariffs on imports.	**2.88**	**0.14**
Had an appendicitis operation.	**2.88**	**0.06**
Increased import tariffs.	**2.92**	**0.13**
Wears blue shirts normally.	**2.93**	**0.05**
Is lactose intolerant.	**2.95**	**0.05**
Prefers juice without pulp.	**2.99**	**0.05**
Does not wear a wristwatch.	**3.02**	**0.06**
Prefers summer to winter.	**3.12**	**0.1**
Prefers android operating system.	**3.15**	**0.06**
Likes dancing tango.	**3.15**	**0.07**
Has two nieces.	**3.15**	**0.06**
Travelled to paraguay.	**3.16**	**0.07**
Watch television series on their days off.	**3.19**	**0.07**
Can play ping pong very well.	**3.21**	**0.07**
Wears reading glasses.	**3.21**	**0.06**
Knows how to cook oriental food.	**3.22**	**0.08**
Increased the number of state jobs.	**3.25**	**0.13**
Likes tomatoes.	**3.25**	**0.08**
Can play the accordion.	**3.27**	**0.07**
Likes listening to the radio.	**3.27**	**0.07**
Likes basketball.	**3.27**	**0.07**
Took a carpentry course.	**3.32**	**0.08**
Their favourite food is pasta.	**3.34**	**0.07**
Likes beekeeping.	**3.37**	**0.08**
Lifted the dollar tax.	**3.38**	**0.13**
Likes singing while taking a bath.	**3.39**	**0.09**
Knows how to ride a bicycle.	**3.4**	**0.09**
Likes visual art.	**3.4**	**0.07**

Read the book martin fierro.	**3.4**	**0.08**
Vacationed in misiones.	**3.41**	**0.08**
Likes ice cream.	**3.41**	**0.1**
Plays football on saturdays.	**3.43**	**0.09**
Likes salads.	**3.45**	**0.09**
Likes to see theatre plays.	**3.47**	**0.09**
Lowered the retirement age.	**3.47**	**0.16**
Likes italian food.	**3.47**	**0.09**
Practices yoga every day.	**3.5**	**0.1**
Likes geting up early.	**3.5**	**0.09**
Sleeps 8 hours a day.	**3.54**	**0.09**
Participated in a community pot.	**3.54**	**0.1**
Likes reading before going to sleep.	**3.56**	**0.09**
Likes going to the cinema.	**3.56**	**0.1**
Is physically active.	**3.59**	**0.09**
Has medical check-ups every year.	**3.61**	**0.1**
Adopted a pet.	**3.63**	**0.08**
Likes plants.	**3.63**	**0.09**
Walks with their dog.	**3.68**	**0.1**
Added subsidies to basic goods.	**3.69**	**0.12**
Has good computer skills.	**3.7**	**0.08**
Paid off debts assumed with the imf.	**3.76**	**0.15**
Has visited national parks.	**3.81**	**0.09**
Listens to classical music.	**3.87**	**0.6**
Is in favour of marijuana legalisation.	**3.9**	**0.11**
Admitted mistakes in their mandate.	**3.94**	**0.11**
Increased restrictions on mining companies.	**3.97**	**0.1**
Donated part of their salary to children's soup kitchens.	**3.97**	**0.1**
Decreased repression while in power.	**4.03**	**0.1**
Lowered taxes.	**4.04**	**0.09**
Accepted criticism from the opposition.	**4.07**	**0.08**
Promoted local tourism.	**4.09**	**0.09**
Is a generous person.	**4.09**	**0.09**
Decreased unemployment while in power.	**4.13**	**0.14**
Carried out a public works plan.	**4.22**	**0.09**
Respectfully debated with the opposition.	**4.26**	**0.09**
Improved the quality of green spaces.	**4.26**	**0.07**
Is in favour of the secular state.	**4.27**	**0.11**
Promoted cultural exchanges.	**4.28**	**0.08**
Accepted salary increases in bargaining processes.	**4.31**	**0.09**
Increased retirement pensions.	**4.31**	**0.08**
Dismissed an official for corruption.	**4.33**	**0.1**
Banned the use of toxic agro-chemicals.	**4.33**	**0.11**
Is in favour of legalising abortion.	**4.38**	**0.09**
Increased the minimum wage.	**4.42**	**0.08**
Improved transport infrastructure.	**4.48**	**0.07**
Encouraged the development of the national industry.	**4.53**	**0.07**
Increased the science budget.	**4.58**	**0.06**

Encouraged environmental policies.	**4.62**	**0.06**
Increased the public health budget.	**4.63**	**0.06**
Reduced poverty while in power.	**4.69**	**0.07**
Increased the education budget.	**4.79**	**0.05**

Supplemental Table 3. Survey Sections and Variables

SECTION 1. PRESENTATION OF THE SURVEY AND INFORMED CONSENT
SECTION 2. PERSONAL CHARACTERIZATION
2.1. GENDER
2.2. AGE
2.3. RESIDENCE STATE
2.4. SOCIO- ECONOMIC SELF-PERCEPTION
2.5. EDUCATION LEVEL
SECTION 3. POLITICAL CHARACTERIZATION
3.1. SELF-PERCEPTION: POLITICAL & APOLITICAL & NEUTRAL PERSON
3.2. SELF-PERCEPTION: LEFT & CENTRE & RIGHT WINGS
3.3. POLITICAL PARTY AFFILIATION (YES/NO)
SECTION 4. SUBJECTIVE VARIABLES ABOUT EACH CANDIDATE
4.1. RECOGNITION (YES/NO)
4.2. TRUST (A 9-POINT LIKERT SCALE)
4.3. FAMILIARITY (A 9-POINT LIKERT SCALE)
4.4. VOTING PROBABILITY (A 9-POINT LIKERT SCALE) *
SECTION 5. VOTE CHARACTERIZATION
VOTE REASONS (PIT: political ideological tradition; CO: candidate opposition; EB: economic benefit; EA: emotional affinity; I: influence; Other)
SECTION 6. MEANS USED TO INFORM ABOUT CANDIDATES
6.1. INFORMATION MEANS (Press Media; Audio-visual Media; Social Media; Campaigns; Mouth to mouth; Other)
6.2. WRITTEN MEDIA (Ambito; Clarin; Pagina 12; Perfil; LID; Prensa obrera; La Nacion; Cronista; Popular; InfoBae; Tiempo Argentino; Scramble; None)
6.3. AUDIO-VISUAL NEWS PROGRAMMES (TV publica; Channel 26; C5N; Channel 13; Cronica TV; Channel 9; America TV; Telefe; TN; Scramble; None)
SECTION 7. ACKNOWLEDGEMENT AND DIFFUSION

*For analysis, Voting Probability was considered as the Response Variable and the others as Explanatory Variables.

Supplemental Table 4. Collected Press Media News

Press Media	Total News	Headlines*	Percentage of reported consultation#	
			PERIOD #1	PERIOD #2
Ambito	1739	241	18.87	21.38
Clarin	4113	412	43.32	46.78
Cronista	1513	268	11.24	14.94
Infobae	5956	374	55.25	61.15
La Izquierda Diario	1275	260	19.92	19.17
La Nacion	4862	393	47.76	52.86
Pagina 12	810	82	52.91	54.57
Perfil	992	70	21.84	29.32
Popular	346	39	7.08	5.36
Prensa Obrera	192	19	6.67	6.43
Tiempo Argentino	712	95	15.63	18.24

**Articles headlines mentioning any presidential formula/candidate.*

#The percentage of reported consultation was extracted from the Subjective Dataset.

Supplemental Table 5. Keywords for Candidate/Presidential Formulas Mention

Candidate target	President Candidate	Vice-president Candidate	Political Force	Other References
AF	Alberto Fernandez; Alberto*	Cristina Fernandez de Kirchner; Cristina*; Cristina Fernandez; Cristina Kirchner; CFK	Frente de Todos	Kirchnerismo
MM	Mauricio Macri; Macri*; Mauricio*	Miguel Angel Pichetto; Pichetto	Juntos por el Cambio	Macrismo, PRO
RL	Roberto Lavagna; Lavagna	Juan Manuel Urtubey; Urtubey	Consenso Federal	-
NDC	Nicolas Del Caño; Del Caño	Romina Del Pla; Del Pla	Frente de Izquierda y de los Trabajadores-Unidad; Frente de Izquierda; FIT	-
JGC	Jorge Gomez Centurion; Centurion	Cynthia Hotton; Hotton	Frente NOS; NOS	-
JLE	Jose Luis Espert; Espert	Luis Rosales	Unite por la Libertad y la Dignidad; UNITE	-

**These keywords were checked to be mentioning the respecting candidate or political force*

Supplemental Table 6. Social Study and election results

Presidential Candidate	PASO election					General Election								
	Fam. (se)	*Trust (se)*	*VP (se)*	*Campaign expenses*	*Votes (%)*	*Fam. (se)*	*Trust (se)*	*VP. (se)*	*EXP_CORPUS (se)*	*EXP_HEAD (se)*	*POS_W (se)*	*NEG_W (se)*	*Campaign expenses*	*Votes (%)*
Alberto Fernandez (AF)	7.21 (0.076)	4.50 (0.083)	4.82 (0.093)	49,022,447	12,205,085 (47.78)	7.82 (0.088)	4.32 (0.105)	4.88 (0.121)	3993.50 (78.612)	374.03 (6.96)	0.53 (0.007)	0.32 (0.004)	79,189,766	12,946,037 (47.46)
Mauricio Macri (MM)	6.01 (0.086)	2.32 (0.069)	2.47 (0.078)	150,950,588	8,121,596 (31.80)	7.75 (0.092)	2.74 (0.101)	3.16 (0.12)	5460.54 (89.943)	516.17 (8.247)	0.25 (0.002)	0.37 (0.004)	142,243,286	10,811,586 (39.63)
Roberto Lavagna (RL)	4.31 (0.066)	2.64 (0.054)	1.56 (0.051)	7,346,177	2,081,293 (8.15)	5.34 (0.078)	3.25 (0.069)	1,69 (0.06)	727.32 (14.69)	48.61 (0.94)	0.64 (0.007)	0.15 (0.003)	18,398,605	1,649,322 (6.05)
Nicolas Del Caño (NDC)	4.91 (0.066)	3.41 (0.064)	1,87 (0.061)	15,624,624	723,128 (2.83)	5.42 (0.085)	3.18 (0.077)	1.67 (0.073)	1008.65 (24.30)	53.70 (1.61)	0.50 (0.01)	0.11 (0.002)	17,160,085	579,228 (2.12)
J.J.Gómez Centurión (GC)	1.53 (0.049)	1.22 (0.047)	0.59 (0.036)	1,466,545	670,156 (2.62)	2.45 (0.066)	1.41 (0.059)	0.44 (0.044)	296.68 (5.84)	16.07 (0.31)	0.10 (0.002)	0.29 (0.004)	11,926,263	457,956 (1.68)
José Luis Espert (JLE)	3.33 (0.07)	2,39 (0.06)	1,52 (0.06)	4,250,000	550,58 (2.16)	3.82 (0.075)	2.01 (0.07)	0.91 (0.056)	270.85 (5.35)	10.94 (0.228)	0.40 (0.006)	0.28 (0.006)	14,120,000	394.207 (1.45)
Manuela Castañeira (MC)*	2.59 (0.059)	2,44 (0.059)	1.16 (0.045)	2,932,818	179,428 (0.70)									
Alejandro Biondini (AB)*	1.231 (0.045)	0.44 (0.026)	0.21 (0.019)	N/A	58,938 (0.23)									
Raúl Albarracín (RA)*#	N/A	N/A	N/A	N/A	36,410 (0.14)									
José A. Romero Feris (RF)*	0.66 (0.029)	0.82 (0.032)	0.21 (0.018)	N/A	32,719 (0.13)									
